# Adhesion and Colonization of the *Probiotic Lactobacillus plantarum* HC-2 in the Intestine of *Litopenaeus Vannamei* Are Associated With Bacterial Surface Proteins

**DOI:** 10.3389/fmicb.2022.878874

**Published:** 2022-04-13

**Authors:** Yang Du, Hao Li, Jianchun Shao, Ting Wu, WenLong Xu, Xiaoman Hu, Jiong Chen

**Affiliations:** ^1^State Key Laboratory for Managing Biotic and Chemical Threats to the Quality and Safety of Agro-products, Ningbo University, Ningbo, China; ^2^Laboratory of Biochemistry and Molecular Biology, School of Marine Sciences, Ningbo University, Ningbo, China; ^3^Key Laboratory of Aquacultural Biotechnology of Ministry of Education, Ningbo University, Ningbo, China; ^4^Key Laboratory of Marine Biotechnology of Fujian Province, Institute of Oceanology, Fujian Agriculture and Forestry University, Fuzhou, China

**Keywords:** *Lactobacillus plantarum* HC-2, *Litopenaeus vannamei*, surface-associated protein, bacterial adhesion and colonization, probiotic

## Abstract

Surface proteins are a type of proteins expressed on the surface of bacteria that play an important role in cell wall synthesis, maintenance of cell morphology, and signaling with the host. Our previous study showed that the probiotic *Lactobacillus plantarum* HC-2 improved the growth performance and immune response of *Litopenaeus vannamei*. To further investigate the probiotic mechanism, we determined the automatic aggregation ability of the bacteria and surface hydrophobicity of HC-2 after being treated with 5 M of lithium chloride (LiCl) and observed the morphology and adhesion of the bacteria to HCT116 cells. The results showed that with the removal of the HC-2 surface protein, the auto-aggregation ability and surface hydrophobicity of HC-2 decreased, and the crude mucus layer coated on the bacterial surface gradually dissociated. The adhesion rate of HC-2 to HCT116 cells decreased from 98.1 to 20.9%. Moreover, a total of 201 unique proteins were identified from the mixture of the surface proteins by mass spectrometry (MS). Several proteins are involved in transcription and translation, biosynthetic or metabolic process, cell cycle or division, cell wall synthesis, and emergency response. Meanwhile, a quantitative real-time PCR qPCR_ showed that HC-2 was mainly colonized in the midgut of shrimp, and the colonization numbers were 15 times higher than that in the foregut, while the colonization rate in the hindgut was lower. The adhesion activity measurement showed that the adhesion level of HC-2 to crude intestinal mucus of *L. vannamei* was higher than that of bovine serum albumin (BSA) and collagen, and the adhesion capacity of the bacterial cells decreased with the extension of LiCl-treatment time. Finally, we identified the elongation factor Tu, Type I glyceraldehyde-3-phosphate dehydrogenase, small heat shock protein, and 30S ribosomal protein from the surface proteins, which may be the adhesion proteins of HC-2 colonization in the shrimp intestine. The above results indicate that surface proteins play an important role in maintaining the cell structure stability and cell adhesion. Surface proteomics analysis contributes to describing potential protein-mediated probiotic-host interactions. The identification of some interacting proteins in this work may be beneficial to further understand the adhesion/colonization mechanism and probiotic properties of *L. plantarum* HC-2 in the shrimp intestine.

## Introduction

Lactic acid bacteria (LAB) is a kind of Gram-positive bacteria that can utilize fermentable sugars to produce large amounts of lactic acid (Ringø and Gatesoupe, 1998). LAB is a common probiotic that exists in a variety of plants, food, and ecological niches, such as fermented dairy products, and is a natural settler in the gastrointestinal tract of humans and animals, mainly including the genera, LAB, *Enterococcus*, and *Bifidobacterium*. As the most important beneficial microbiome, LAB has been widely used in the food industry, medical care, feeding fermentation, and disease prevention and control of animals (Perdigon et al., 1988; El-Nezami et al., 2002; Roberfroid et al., 2010). Lactobacillus colonizes the gastrointestinal tract, which can enhance the absorption of nutrients of organisms, inhibit the invasion of pathogenic bacteria, fight against tumors, and reduce cholesterol (Chan et al., 1985; Bernet et al., 1993; Hooper and Gordon, 2001). In the process of fermentation, LAB produces some bacteriostatic substances, such as streptococcus, lactobacillus, and acidophilus, which can inhibit the growth of putrefactive bacteria or pathogenic bacteria. It is best to take antibiotics along with LAB, because in addition to killing pathogenic bacteria, antibiotics also kill the normal flora in the gastrointestinal tract, leading to an imbalance in the micro-ecological balance of the gastrointestinal tract, which also leads to bacterial resistance. However, LAB can promote t he establishment of normal microflora in the gastrointestinal tract of animals, enhance the immunity of animals, improve the efficiency of feed utilization, and improve the quality of animal products by adjusting the balance of micro-ecology of the body, which has remarkable economic and social benefits.

Adhesion is a specific interaction between the bacterial surface components and the complementary structure of the host cell surface, which is a crucial and prerequisite step for bacterial colonization in the digestive tract (Buck et al., 2005; Bagon et al., 2018; Montoro et al., 2018). Several adhesion factors have been identified which facilitate binding to mucosal and epithelial surfaces and facilitate colonization or the formation of vegetation (Koch et al., 2004). Among the adhesive components, surface proteins have been proposed to be involved in the colonization of gastrointestinal epithelial cells and mucosa of mammals (Rojas et al., 2002). The outermost surface-layer (S-layer) protein is composed of a single protein or glycoprotein subunit with specific biofilm structures, which are almost ubiquitously present in the surface of all archaea and some gram-positive and gram-negative bacteria, involving more than 300 species (Sára and Sleytr, 2000; Pavkov-Keller et al., 2011). While the S-layer proteins are the most abundant proteins produced by cells, there is a wide diversity in their sequence and function, displaying oblique, square, or hexagonal symmetries (Engelhardt, 2007). These proteins bind to the outermost layer of the cell with non-covalent bonds, using denaturants, such as lithium chloride (LiCl), guanidine hydrochloride (GuHCl), urea, or metal chelating agents, etc., which can depolymerize the S-layer proteins to monomers. When the denaturant is removed, the proteins subunit can be reassembled into regular crystals in a variety of substrates, that is, they occur as self-assembly (Sleytr, 1997; Sleytr et al., 2001). Several lactic acid bacteria have S-layer proteins on their surfaces, and some strains may have more than one S-layer protein on their surfaces (Sára and Sleytr, 2000), and the probiotic function of LAB S-layer protein has been reported recently, but it is not clear enough (Jakava-Viljanen and Palva, 2007).

The gastrointestinal tract is naturally inhabited by a large number of commensal flora, some of which are beneficial to the host, while others are potential pathogens. The characterization of the surface-associated proteins contributes to a better understanding of endosymbiotic bacteria-specific adaptation and interactions with the host in the gastrointestinal tract and pathogen transmission. Over the past years, the extracellular proteins of several gram-positive bacteria have been identified and analyzed by proteomics methods. Two-dimensional electrophoresis and mass spectrometry (MS) were usually employed to analyze the protein extraction from cell wall fractions of culture supernatants (Antelmann et al., 2001; Cole et al., 2005; Voigt et al., 2006; Mueller et al., 2007). Recently, the development of *in situ* protein analysis techniques allows for accurate and rapid identification of protein species and relative expression from mixed samples according to “shaving” the surface of the intact cell using denaturants or enzymes; then the enzymatically digested short peptides were analyzed by liquid chromatography (LC) and tandem mass spectrometry (MS/MS) (Rodríguez-Ortega et al., 2006). In our previous work, the *Lactobacillus plantarum* HC-2 strain was isolated from aquatic animals (*Chaeturichthys stigmatia*), which has high antibacterial and adhesive activities to the intestinal mucus of *Litopenaeus vannamei*. Additionally, the diet experiment demonstrated that HC-2 is a probiotic strain that could improve the immune responses, growth performance, bacterial composition, and disease resistance of the *L. vannamei* (Sha et al., 2016a), but the probiotic mechanism is still not definitively or completely understood. Therefore, exploring the mediate-function of the surface proteins to the adhesion colonization and immunomodulatory effect of *L. plantarum* HC-2 in the intestine of *L. vannamei* is required.

In the present study, we have applied the “shave off” approach with 5 M- LiCl to isolate the surface proteins of *L. plantarum* HC-2, and further identified those proteins using MS. Scanning electron microscope (SEM) and electron microscopy were used to observe the morphology of bacterial cells before and after LiCl-treatment, and then explored the renewable characteristics of surface proteins. We investigated the effect of surface proteins on the adhesion rate of HC-2 to HCT116 cells and shrimp intestinal mucus. The aim is to characterize the biological properties of *L. plantarum* HC-2 and provide a theoretical basis for its application and promotion in shrimp culture.

## Materials and Methods

### Bacteria and Culture Conditions

*Lactobacillus plantarum* HC-2 (GenBank No. CP046935; BioProject No. PRJNA579442) was isolated from the intestine of *Chaeturichthys stigmatias* and stored in de Man, Rogosa, and Sharpe (MRS) broth (Luqiao Biotechnology Co., Ltd, Qingdao, China) containing 20% (v/v) of glycerol (Sha et al., 2016b). The strains were removed from −80°C and revived, then inoculated in fresh MRS broth medium at a ratio of 1:100 and expanded at 37°C under sealed anaerobic resting conditions for 24 h.

### Isolation and Extraction of Surface Proteins

Surface protein extracts were prepared according to the method described by Zhang et al. (2013) and Singh et al. (2016). The bacterial growth density was measured after approximately 20 h of incubation, and the bacteria were collected at the time when the bacterial growth index phase reached a plateau (OD600 ≈ 1.7). About 1 L of the culture volume was centrifuged (3,000 g, 10 min, 4°C), and the supernatant was discarded to collect the precipitate, which was then washed three times with 1 M of sterile phosphate-buffered saline (PBS) buffer, and the precipitate was resuspended in a PBS containing 25% of sucrose. One milliliter of 5 M LiCl per 25 mg wet weight of bacteria was added, and 0.64 mL of Sigma Protease inhibitor (Sigma-Aldrich, St. Louis, MO) were added and incubated at 37°C with shaking at 180 rpm for 3, 6, 9, and 15 h. After treatments, cells were centrifuged (13,000 g, 10 min) and the supernatant was filtered through a 0.22 mm aqueous membrane (Sartorius AG, Goettingen, Germany). The collected supernatant samples were loaded into dialysis bags with an MW of 3 kDa, then placed in pure water containing protease inhibitors at 4°C for dialysis, and the dialysate was changed every 4 h, 4 times in total. After dialysis, the dialysis samples were freeze-dried for 48 h. The crude samples were dissolved in a protein buffer (7M of urea, 2M of thiourea, 4% of CHAPS, 20 mM of Tris, pH 8.5), and the protein concentration was measured using the bicinchoninic acid (BCA) Protein Assay kit (Pierce, Rockford, IL, USA). Protein samples were boiled in a loading buffer and separated by sodium dodecyl sulfate-polyacrylamide gel electrophoresis (SDS-PAGE) at a final polyacrylamide concentration of 15% (w/v). The pretreated (i.e., resuspended in sterile PBS) and LiCl-treated bacteria were appropriately diluted and coated on MRS solid medium and incubated at 37°C for 24 h. The cell viability was assessed by counting the number of colonies on the plates.

### Analysis of Auto-Aggregation Ability and Surface Hydrophobicity of HC-2

The automatic aggregation ability of the bacteria and the surface hydrophobicity of HC-2 were determined according to the method of Chen (2009) with appropriate changes. The concentration of the bacterial suspension was adjusted to 10^8^ CFU/mL. The upper layer bacterial suspension of 2 mL was mixed for 15 s and placed at room temperature. The absorbance at 600 nm was determined. The formula for calculating the percentage of auto-aggregation is as follows:

auto-aggregation ability/% = (1-An/A0) × 100

where A0 represents the absorbance of the initial bacterial suspension at 600 nm, and An represents the absorbance of the bacterial suspension at 600 nm after different LiCl-treatments.

According to the method described by Kos et al. (2003), the absorbance of a bacterial suspension at 600 nm, was determined by adjusting the concentration of the bacterial suspension to 10^8^ CFU/mL, which was marked as A0. About 3 mL of adjusted concentration of bacterial liquid was taken, and 1 mL of xylene was added into it. The suspension was shaken for 5 min, allowed to stand for 30 min, and waited for delamination. The aqueous phase was taken and A1 was determined at 600 nm. The formula for calculating the surface hydrophobicity of bacterial cells is as follows:

surface hydrophobicity/% = (1-Am/A0) × 100

where A0 represents the absorbance of the initial bacterial suspension at 600 nm, and Am represents the absorbance of the aqueous bacterial suspension at 600 nm after different LiCl-treatment.

### Electron Microscope Observation on the Shape and Structure of HC-2

The appearance characteristics before or after the treatment of *L. plantarum* HC-2 strain with LiCl were examined using a SEM according to the methods described by Nyenje et al. (2012) with some modifications. For this, 1 mL of bacterial suspension was taken (10^8^ CFU/mL) in 1.5 mL of sterile stub, 3,000 g for 10 min; then the bacterial body was washed twice with 1 mL of sterile PBS (0.01 mol/L pH7.4) and 2.5% glutaraldehyde fixation was added to it (refrigerated at 4°C overnight). Further, the bacteria were centrifuged and washed 3 times with PBS, each time for 15 min, and the samples were fixed with 1% of osmium acid for 1 h, rinsed 3 times with 0.1 M of PBS (15 min/time), then dehydrated in 30, 50, 70, 80, 90, 95, and 100% ethanol (15 min for each concentration), exposed to tert-butanol replacement, and placed at −80°C for overnight freezing; the samples were freeze-dried for 24 h, and sputtered with Elko 1B for gold-palladium plating. The samples were finally observed with an SEM (Model: Hitachi S-3400N).

Samples of *L. plantarum* HC-2 strain before or after being treated with LiCl were characterized by transmission electron microscopy (TEM). First, the surface structure of the bacteria was observed by negative staining as described by Mörgelin et al. (1992). The bronze grids were put into the glow discharge device to perform hydrophilic glow discharge on it for 30 s. Then, the grids were put onto the surface of 10^8^ CFU/mL of bacterial solution, so that the sample was adsorbed onto the grids, rinsed twice with sterile PBS, and then stained by touching the surface of different drops for 60 s, respectively. Finally, the grids were put onto a filter paper to absorb the water and dried naturally. Additionally, the internal structure of the bacteria was observed by the ultrathin sections as described by Garrote et al. (2004). For thin sections, the cells were fixed with 20 g of glutaraldehyde (PBS buffer, pH 7.2) for 15 h at room temperature. The cells were collected by centrifugation (3,000 g, 10 min) and washed 3 times with sterile PBS. The samples were then fixed in a phosphate buffer containing 10 g of osmium tetroxide and dehydrated for 2 h. The samples were embedded in Epon 812, and ultra-thin sections were prepared using a Sorvall MT 2B ultramicrotome. The samples were then negatively stained with 0.1 m of ammonium acetate buffer containing 10 g of ammonium molybdate, pH 7.0 (Masuda and Kawata, 1980), and finally observed using a TEM (at JEOL 1200-EXII, Japan).

### Microscopic Observation and Flow Cytometry Analysis

For prelabel, the bacteria obtained before and after LiCl treatment were resuspended in a sterile PBS buffer, and the cell density was adjusted to 1 × 10^9^ CFU/mL. Fluorescein isothiocyanate (FITC) stock solution was added to the bacterial resuspension to reach a final concentration of 20 μmol/L. The bacteria were incubated for 1 h at 37°C with shaking under dark conditions, and the uncoupled FITC was then removed by washing 3 times with sterile PBS (12,000 g, 4°C, 3 min). The bacteria were resuspended in sterile PBS and fixed with formaldehyde at a final concentration of 0.75% (v/v) for 2 h. The labeling rate was then analyzed using a flow cytometer (BD FACSAria™II, USA) to confirm that the stability of the fluorescence met the experimental requirements.

The HCT116 cells were taken out from the liquid nitrogen and quickly melted in a water bath at 37°C. Under aseptic conditions, the medium liquid was sucked into a 10 mL sterile centrifuge tube, and a 3 mL of high-sugar DMEM medium was added. Centrifugation was performed at 4°C at 1,000 g for 5 min, and the supernatant was discarded. About 5 mL of high-sugar DMEM medium containing 10% of heat-inactivated fetal bovine serum and 1% of double antibody (penicillin concentration 100 U/mL, streptomycin concentration 100 μg/mL) were added. After being gently blown and mixed, the cells were transferred to a 50 mL cell culture flask and cultured in a 5% CO_2_ incubator at 37°C. The high-sugar DMEM medium was replaced with a fresh medium every other day. When the cells were growing well (the coverage rate of monolayer adherent cells reached about 80%), 1 mL of 0.25% trypsin-EDTA was used for digestion and passage. After 8 or 10 times of passage, the cells become polarized, and the adhesion test was carried out.

After being labeled with FITC, the bacteria were washed with sterile PBS 5 times to remove the unlabeled FITC. One day before the test, a high-sugar DMEM medium without double antibodies was added to the 6-well plate. Before adding bacteria, single layer HCT116 cells were washed with sterile PBS well plate twice, and 500 μL of bacteria with a concentration of 10^8^ CFU/mL were added to each well. The 6-well plates were transferred to a 5% CO_2_ incubator and cultured at 37°C for 2 h. The monolayer cell layer was washed more than thrice with PBS to elute unattached bacteria and metabolic secretions. The cell slides were taken out and photographed under a fluorescence microscope. In addition, 250 μL of 0.25% of trypsin-EDTA was added to each well and incubated for 10 min. Then 250 μL of serum was added to stop digestion. The solution in each well was collected and detected by flow cytometry. In addition, another portion of the cell slides was fixed with 10% of formalin, and finally, the cells were stained by the hematoxylin & eosin (HE) staining method and observed under a 40-fold microscope.

### MS and Protein Identification

The preparation of protein fraction peptides was done according to the method by Guillot et al. (2003) with some modifications. Bands were excised from gels and were sliced into 1–2 mm^2^ pieces. Each small gel was digested with 0.1 μg of trypsin in 25 μL of 25 mM ammonium bicarbonate (400 g, 37°C) for 12 h. The trypsin reaction was terminated by the addition of 0.1% of formic acid. The supernatant was transferred to a new centrifuge tube, and the remaining fraction was the component containing the peptide. The peptide was extracted by adding 0.1% (v/v) of trifluoroacetic acid containing 60% (v/v) of acetonitrile (400 g, 37 °C) and incubated for 15 h. Finally, the peptide was dried in a freeze-dryer and dissolved in 30 μL of 0.1% (v/v) TFA.

Peptide samples were efficiently desalted with C18 stage tips (Rappsilber et al., 2003) before MS analysis (UltraFleXtreme III MALDI-TOF/TOF mass spectrometer, Bruker Daltonics, Germany). The MS analysis was done by a Thermo Q-Exactive type mass spectrometer. The MS mode PMF spectra were acquired in the range of 600–4500 da using a standard TOF-MS protocol, and the MS mode was acquired in the MS/MS mode using a standard TOF-MS /MS protocol. The ion source parameters were set as follows: spray voltage: 2.1 kV; capillary temperature: 250°C; ion source: EASY-spray source; DP: 100; the raw files of the generated mass spectra were processed using FlexAnalysis software 3.0 (Bruker Daltonics) software.

### Bioinformatics Analyses of Proteins

The MS results were imported into the Uniprot database for search and comparison analysis Mascot Distiller (BioTools v3.1 connected to Mascot, Version 2.2.04, Matrix Science) as described earlier (Anaganti et al., 2015). The Mascot search parameters were set as follows: parent mass error tolerance of 10.0 ppm, fragment mass error tolerance of 0.02 Da; trypsin was used for enzyme cleavage, fixed carbamidomethyl alkylation of cysteines and methionine oxidation were used as variable modification. The target protein was identified as the same protein as the retrieved protein when two of the following three criteria were met: MASCOT search score >60 (*p* < 0.05), at least 5 peptide matches, and >20% amino acid sequence coverage.

Functional analysis based on the association of Gene Ontology (GO) terms to genes in a selected proteins list were applied to the identified proteins using Blast2GO (Götz et al., 2008) and AmiGO 2.0 version (http://amigo.geneontology.org/amigo). The macroscopic statistical analysis was done using the annotation of GO Slim as described in the next section. Blast comparison and annotation were performed using default parameters. Finally, the enriched results were aggregated into a histogram using Blast2GO sorted by 3 secondary levels: molecular function, biological processes, and cellular components. The sequences of all identified proteins were analyzed using several bioinformatics tools to verify or to adjust the localization given in the database, including the Signal P 3.0 server (Bendtsen et al., 2004) and LipoP v 1.0 (Juncker et al., 2003) were used to predict the putative N-terminal signal sequences and cleavage sites. The TMHMM Server v. 2.0 (Krogh et al., 2001) was used to predicting multiple transmembrane helix structures or N-terminal transmembrane anchoring motifs in proteins, and the SecretomeP 2.0 Server (Bendtsen et al., 2004) was used to predict the proteins with features indicating non-classical secretion. The colonization rate was detected in the intestine.

### Diets Preparation, Feeding Trials, and Bacterial Colonization Assay

A total of 200 healthy shrimps (weight 3.5 ± 0.06 g) were obtained from the Xianxiang aquaculture base (Ningbo, China) and were randomly divided into the 5 aquaria. The shrimps were domesticated with basal feed for 1 week before starting the experiment. The feed was prepared as follows; the bacteria untreated or treated by LiCl bacteria were labeled with FITC according to the method described in Section Microscopic observation and flow cytometry analysis, and resuspended in sterilized seawater. The bacterial suspension was evenly sprayed on the basic commercial feed with the final concentration reaching 5 × 10^8^ CFU/g, and then placed at room temperature for 4–5 h to dry and finally stored at 4 °C. The shrimp culture temperature was maintained at 30 ± 2°C, seawater salinity was adjusted to 30%, continuously oxygenated, and 25% (v/v) water changes per day. The feeding rate was 35, 20, and 45%, three times a day, and the daily feeding rate was controlled at 10% of body weight. The feeding experiment lasted for 2 weeks, and then starved for one day. Three shrimps from each group were taken, and the intestinal contents were washed with sterile PBS. The intestinal contents were longitudinally sliced with a sterile scalpel and then fixed on slides for observation under a fluorescence microscope. In addition, six shrimps were taken from each group for intestinal bacteria detection, as described in a previous study (Du et al., 2019). Briefly, a quantitative real-time PCR (qPCR) standard curve was constructed to quantify the number of HC-2 using *L. plantarum*-specific primers (F: 5′-TTATTCAAGCCGTCGGAGTG-3′; R: 5′-TCGCTGGTGCTAATGCAATG-3′.) with 128 bp amplicon (Kim et al., 2020). Shrimp intestinal tissue DNA was extracted using a marine animal tissue DNA extraction kit (Qiagen, Germany), and the sample concentration was adjusted to 100 ng/μL. RNase-free water was used as a negative control for the qPCR assay. The qPCR reaction system was 20 μL, containing 2 μL (100 ng/μL) of DNA, 10 μL of SYBR Green (Roche, Sweden), 1 μL of primer (10 μM), and 7 μL of RNase-free water. The cycling protocol was as follows: 94°C for 5 min, 45 cycles, denaturation at 94°C for 30 s, and annealing for 30 s. Denaturation at 94°C for 30 s, annealing at 60°C for 10 s, and extension at 72°C for 10 s. DNA melting curve analysis ensured that the desired amplicons were detected and were specific. When the Ct value (y) was obtained, the x value (corresponding bacterial account) was calculated according to the constructed standard curve equation,

y = −3.238 x + 35.346 (R^2^ = 0.9912).

### Detection of Adhesion Rate

The preparation of crude mucus as previously described by Sha et al. (2016a) was done by carefully scraping the intestinal mucous of *L. vannamei* and suspended in precooling HEPES-Hanks's Buffer (Krogh et al., 2001), then centrifuged (12 000 g, 4 °C, 10 min) to remove the insoluble substances. The concentration of crude extracts of intestinal mucus was determined using the Bradford method, and the final concentration was adjusted to 1 mg/mL with HEPES-Hanks's Buffer.

For adhesion testing, 100 μL of prepared shrimp mucus was added into each well of a96- well microtiter plate and incubated overnight at 4°C. Then, the non-immobilized mucus were removed and washed three times with sterile PBS. Subsequently, the labeled bacteria (100 μL) were added to the wells and incubated at 37°C for 1 h. Then, using sterile PBS, the plate was washed three times and the bacteria were liberated by treated with 0.05% of trypsin (100 μL) at room temperature for 10 min. After the reaction, a prechilled fresh brain heart infusion broth (BHIB) medium was added to inactivate the trypsin, and the fluorescence absorption peaks of the samples were measured using a fluorescence spectrophotometer (Hitachi F-4500, Japan) with an excitation wavelength of 500 nm and a slit width of 2.5 nm. The adhesion rate was expressed as the ratio of the fluorescence value of the adherent bacteria to the fluorescence value of the initial bacteria. The ratio was finally used to evaluate the adhesion of *L. plantarum* HC-2 to the crude mucilage. Collagen (Sigma, Missouri, USA) and bovine serum albumin (BSA) (Sigma, Missouri, USA) were used as specific and non-specific adhesion controls, respectively.

### Extraction of Membrane Proteins

Membrane proteins from the intestinal epithelial cells of the shrimp were extracted according to the instructions of the Membrane Protein Extraction Kit (Sangon Biotech Co., Ltd. China). The shrimp intestines were picked out with sterile forceps; the foregut and midgut were intercepted, and the intestinal contents were thoroughly rinsed with a sterile precooled PBS buffer. About 1 mL of an extraction buffer was added to the above tissues and homogenized with a glass homogenizer for 30–50 times. After 10 min on ice, the tissues were centrifuged at 4°C at 14,000 g for 10 min. Then the supernatant was transferred to a new centrifuge tube and placed in a water bath at 37°C for 10 min. The samples were centrifuged at 13,000 g for 5 min at room temperature and then divided into upper and lower layers (containing membrane protein). The bottom layer was removed, 500 μL of cold sterilizing water was added, and placed in an ice bath for 5 min, and at 37°C water bath for 10 min. The samples were centrifuged at 13,000 g for 5 min, and the membrane proteins were collected from the underlying aqueous phase and were saved at −80°C for later use.

### B-NHS Treatment of the Membrane Proteins and the Mucus of the Shrimp

The surface proteins of HC-2 and the membrane proteins, the mucus of the shrimp was labeled with NHS-Biotin kit. The incorporation degree of biotin labeling depends on the amount and concentration of the proteins to be labeled. First, 2 mg of B-NHS was added into 1 mL of diformylamide. After being fully dissolved, the surface protein and the membrane protein, mucus, were respectively adjusted to a final protein concentration of 1 mg/mL in a volume of 10 mL and incubated on ice for 3 h. The treatment solution was placed in the dialysate (10 mmol/L carbonate buffer containing 1.76% of NaCl at pH 9.5) in a refrigerator at 4°C. After full dialysis, the excessive unreacted substances and hydrolyzed biotin reagent were removed through the desalination column, and an equal volume of glycerin was added and stored at −20 °C for later use.

### Identification of HC-2 Surface Adhesion Proteins in the Shrimp Gut

For the identification of HC-2 surface adhesive proteins, the surface proteins were first adjusted to the appropriate concentration and then separated by 15% of SDS-PAGE gel electrophoresis, and then were transferred into a PVDF membrane by wet transfer electrophoresis. After that, the PVDF membrane was sealed overnight with 5% of skim-free milk PBST at 4°C, and the PVDF membrane was rinsed 3 times with PBST, soaked for 5 min each time to wash away the fully bound protein. Then, an appropriate amount of NHS-labeled shrimp intestinal membrane protein and mucus were respectively added for incubation at 37°C for 4 h, and washed with PBST for three times; then 1:1000 AP-conjugated -avidin was added for incubation at 37°C for 2 h, and washed with PBST for three times. The unbound Avidin was washed off, and the PVDF film was finally soaked in the prepared color solution to create color in the dark place. During the process, the color of the solution was monitored, and the reaction was stopped after the desired strip color was obtained. After the PVDF membrane was dried on the filter paper, the picture was photographed.

The identification method of adhesion receptor proteins in the intestines of shrimps was similar to that of adhesive proteins. The intestinal membrane proteins of prawn were adjusted to an appropriate concentration for SDS-PAGE gel electrophoresis, and then the proteins were transferred to the PVDF membrane and incubated with NHS-labeled HC-2 surface proteins, followed by AP-conjugated Avidin. The second antibody labeled with AP was added and incubated.

For the identified positive bands, the corresponding adhesive strips were cut with a sterile surgical blade and then analyzed by MS as described in Section MS and protein identification.

### Statistical Analysis

One-way ANOVA was performed on the repeated measured data using SPSS 23.0 statistical software, with *P* < 0.05 indicating significant differences and *P* < 0.01 indicating highly significant differences. Data were expressed as mean ± standard error (SE). The images were analyzed and plotted using origin 8.0 software and GraphPad Prism 8 software.

## Results

### Extraction of the *L. plantarum* HC-2 Cell Surface Proteins

The *L. plantarum* HC-2 cell and the isolated surface proteins were visualized by Coomassie brilliant blue R-250-staining after 15% of gel were separated by SDS-PAGE. Electro-chorogram showed that several distinct protein bands, some dominant and some less distinct protein bands, were detected in the *L. plantarum* HC-2 cell extracts (Supplementary Figure S1). After the normal bacterial suspension was boiled with protein lysis buffer, almost no protein bands were observed from the SDSPAGE electrophoresis graph, indicating that the protein was not shed from the bacterial surface. While the protein bands could be clearly observed after LiCl treatment. The extraction level of surface proteins was increased in the cells treated by LiCl from 3 to 9 h but was not different from 9 to 15 h. The results indicated that surface proteins were efficiently and selectively extracted by treatment with LiCl for 9 h. Additionally, the surface proteins were remarkably regenerated from the LiCl-treated cells.

### The Automatic Aggregation and Surface Hydrophobicity of HC-2

**Figure 1:**
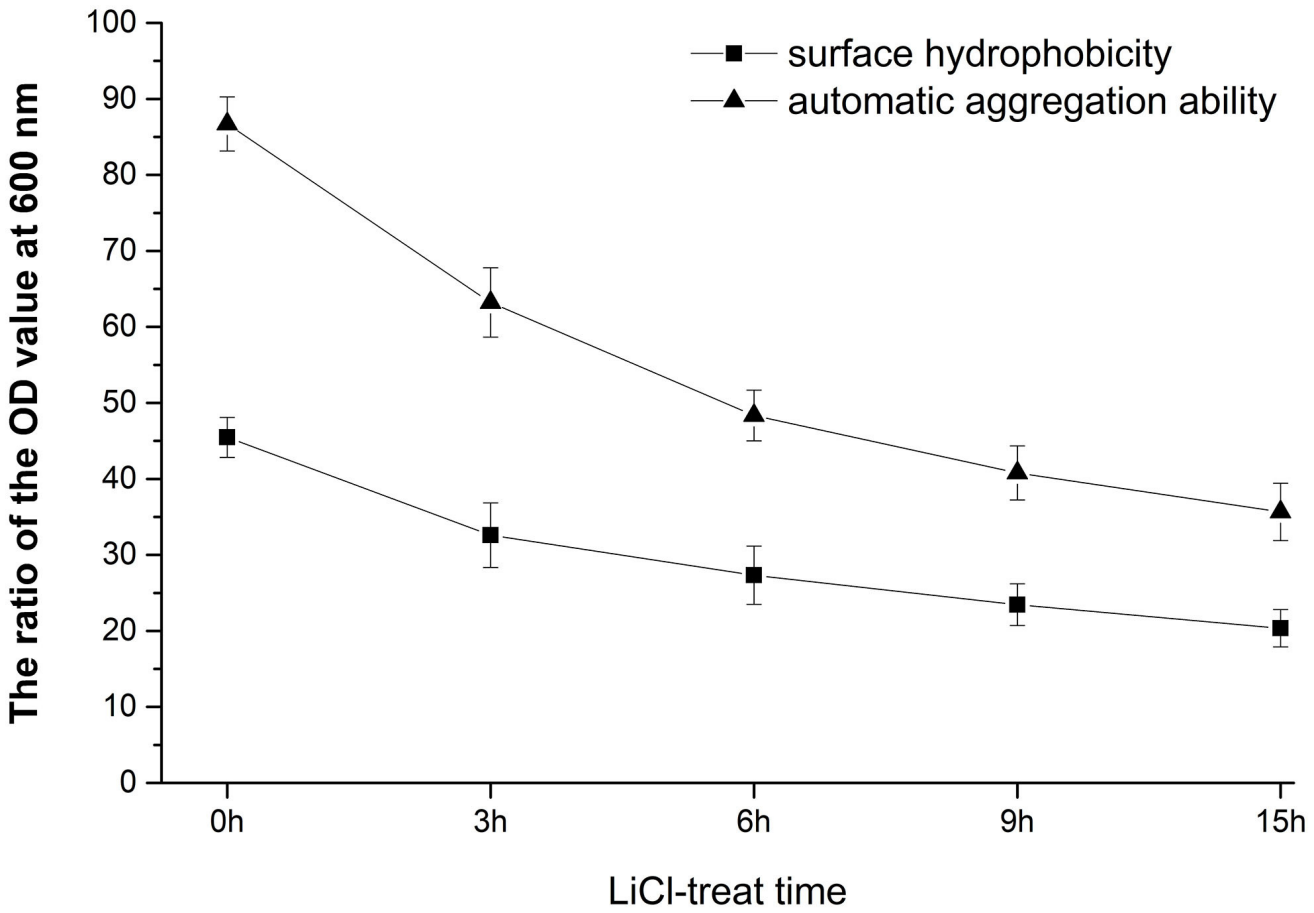
Analysis of surface properties of HC-2 strain. After being treated with lithium chloride (LiCl) at different times, the automatic aggregation ability and surface hydrophobicity rate were decreased.

As can be seen from Figure 1, the longer the treatment time of LiCl, the more proteins are removed from the surface of bacteria, and the self-coagulation rate and surface hydrophobicity of the cells also decrease accordingly. After 9 h of treatment, the decrease in the self-coagulation rate and surface hydrophobicity of the cells is not significant. The results showed that the surface protein had positive effects on the self-coagulation rate and surface hydrophobicity rate of *L. plantarum* HC-2.

### Morphological Characterization of the *L. plantarum* HC-2

**Figure 2:**
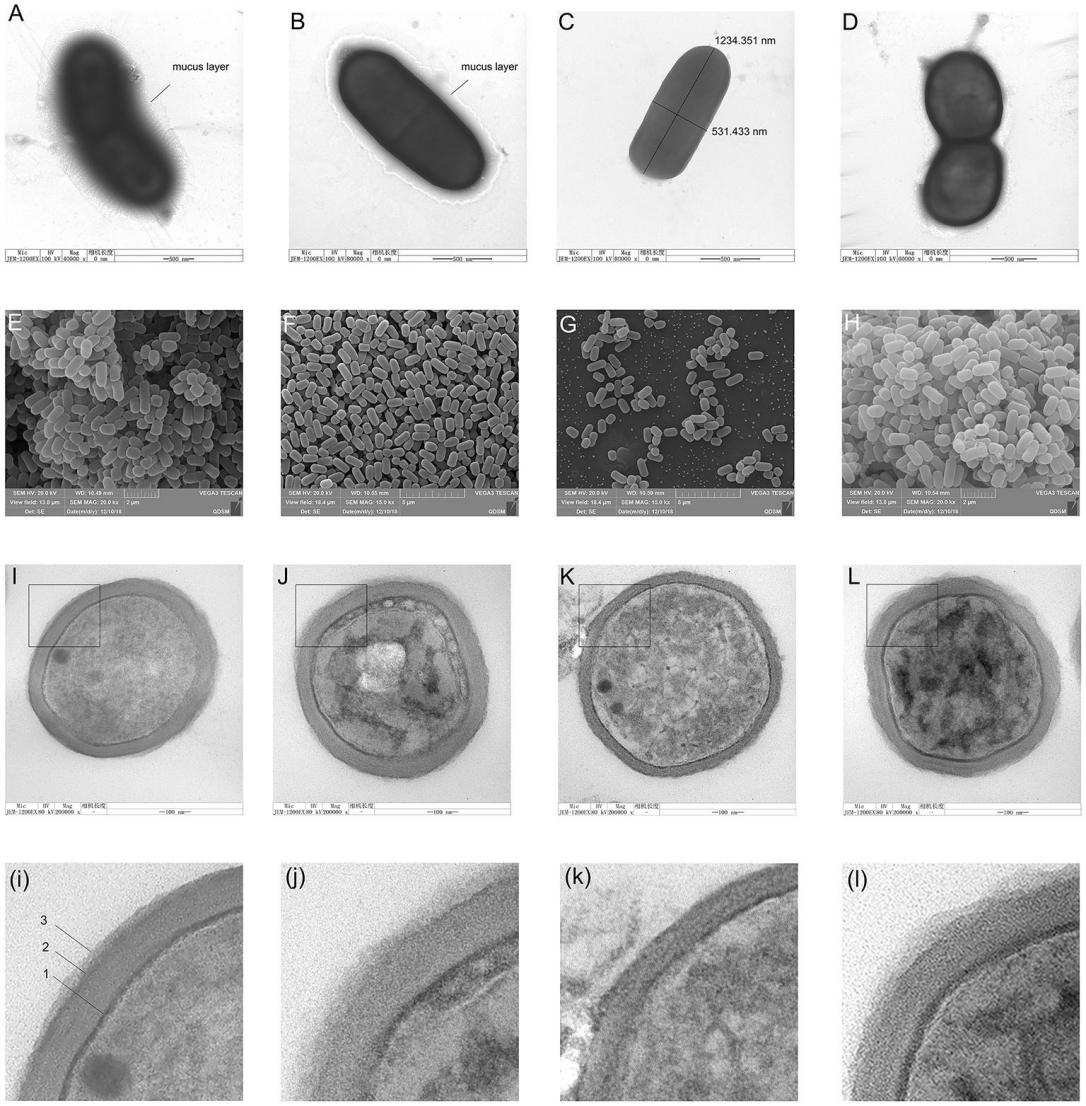
The morphological changes of cells were observed by negative staining electron microscopy, scanning electron microscopy (SEM), and transmission electron microscopy (TEM). Figures from **(A–D)** shows the negative staining with uranyl formate on the carbon support films of *Lactobacillus plantarum* HC-2. Bacteria are adsorbed on the surface of carbon support films from aqueous suspensions containing typically 10^8^ CFU/mL and subsequently stained with 0.75% of uranyl formate solutions. **(A)**, intact *L. plantarum* HC-2. **(B)**, *L. plantarum* HC-2 was treated with LiCl for 3 h. **(C)**, *L. plantarum* HC-2 was treated by LiCl for 9 h. Figures from **(E–H)** show the view of *L. plantarum* HC-2 through SEM. **(E)**, intact *L. plantarum* HC-2 showing the bacterial aggregation. **(F)**, *L. plantarum* HC-2 was treated with LiCl for 3 h showing that the cells were separated. **(G)**, *L. plantarum* HC-2 was treated with LiCl for 9 h. Figures from **(I–L)** show the view of *L. plantarum* HC-2 through TEM. **(I)**, intact *L. plantarum* HC-2 showing a three-layered structure on the cell wall (arrows: 1, plasma membrane; 2, cell wall; 3, the S-layer). **(K)**, *L. plantarum* HC-2 was treated with LiCl for 9 h showing that the cell wall has become thin and the outermost layer is missing. **(L)**, *L. plantarum* HC-2 was treated with LiCl for 9 h and reproduced surface proteins. The graphs of i, j, k, and l are a partial magnification of I, J, K, and L.

The negative staining results showed a thick mucus layer coated on the surface of normal *L. plantarum* HC-2 (Figure 2A). With the extension of LiCl-treatment time, the mucus layer was destroyed (Figure 2B), and after LiCl-treatment for 9 h, the mucus layer on the surface of the bacteria was completely detached (Figure 2C).

Scanning electron photomicrographs of the whole cells of *L. plantarum* HC-2 revealed characteristics of a rough after being treated by LiCl, the refractive index of the bacteria becomes low (Figures 2D–F). After LiCl-treatment, the bacterial cells became more decentralized (Figure 2G), but the ability of adhesion for each other increased after the surface proteins were reformed (Figure 2H).

The TEM ultra-thin sections of *L. plantarum* HC-2 showed a clear three-layer structure on the surface of the bacterium (Figure 2I). (i) The innermost layer is the cytoplasmic membrane, (ii) the middle layer is composed of peptidoglycan, and (iii) the outermost layer is the surface layer, which exhibits a “fuzzy” morphology with an uneven thickness, with the thinnest region being about 9 nm thick and the thicker region being approximately 25 to 35 nm. After treating *L. plantarum* HC-2 with 5 M lithium chloride for 3 h, TEM showed that the surface protein layer was still clearly visible on the surface of the bacteria (Figure 2J), while after 9 h of treatment, the bacterial cell wall thinned and the surface protein layer disappeared completely (Figure 2K). Surface proteins can reform after the LiCl-treated cells are incubated in fresh MRS broth overnight, but the morphological changes were still there (Figure 2L).

### The Adhesion Characteristics of *L. plantarum* HC-2 to HCT116 Cells

**Figure 3:**
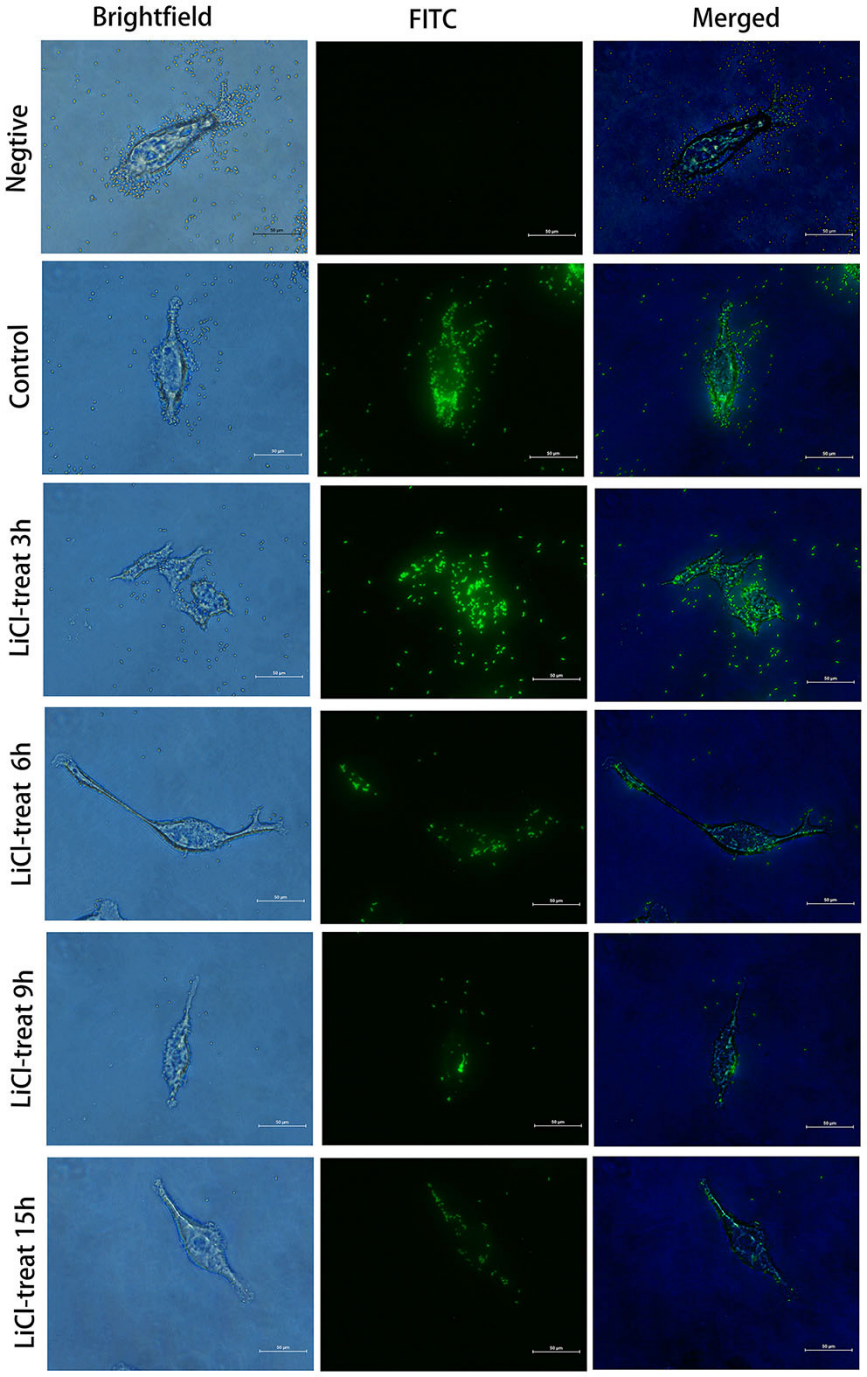
Fluorescence microscope observation of the HCT116 cells adhesion by *L. plantarum* HC-2. After being labeled with fluorescein isothiocyanate (FITC), the bacteria were washed with sterile phosphate-buffered saline (PBS) 5 times, and the concentration of the bacteria was adjusted to 10^8^ CFU/mL and added into the cell culture well. After 2 h of incubation, the cells were washed with sterile PBS 3 times. Then, the cells were fixed with paraformaldehyde, and observed under the fluorescence microscope.

**Figure 4:**
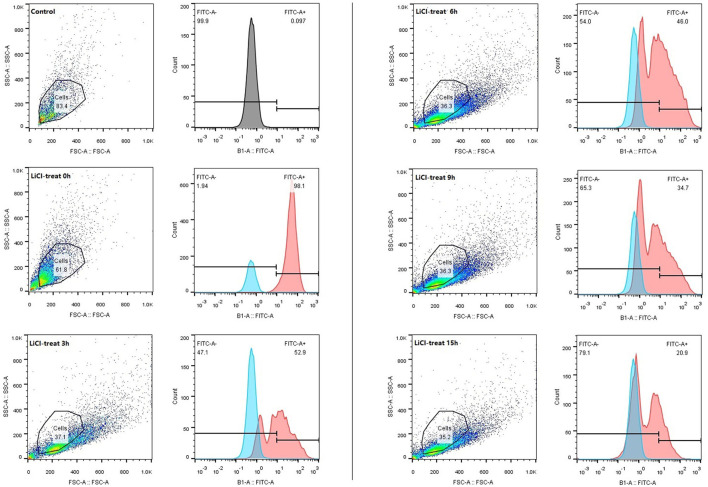
The adhesion rate of the *L. plantarum* HC-2 to the HCT116 cells was analyzed by flow cytometry. After being labeled with FITC, the bacteria were washed with sterile PBS 5 times, the concentration of the bacteria was adjusted to 10^8^ CFU/mL and added into the cell culture well. Incubation was terminated after 2 h. The bacteria were washed with sterile PBS 3 times, and 250 μL 0.25% of trypsin-EDTA was added into each well and incubated for 10 min. About 250 μL serum was added to stop digestion. Cell suspensions were collected and examined by flow cytometry.

**Figure 5:**
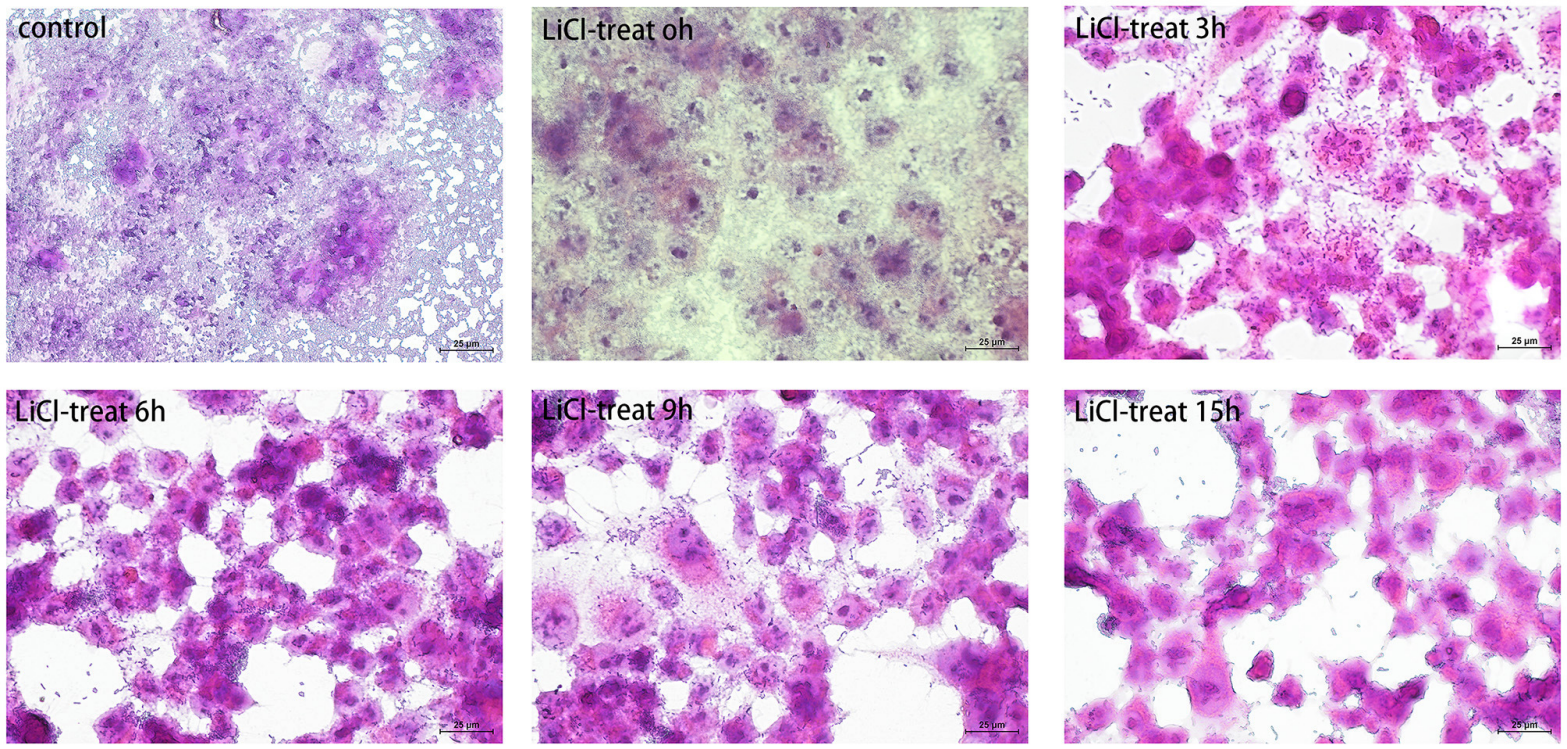
The cells were observed under a microscope by hematoxylin and eosin (HE) staining. The *L. plantarum* HC-2 was treated by LiCl and then incubated with HCT116 cells for 2 h. After incubation, the cells were washed with sterile PBS to remove unattached bacteria. Later, the cells were fixed with 10% formaldehyde, and finally, the cells were stained by the HE staining method and observed under a 40-fold microscope.

The HCT116 cell model is a human-cloned colon adenocarcinoma cell. The structure and function of HCT116 cells are very similar to those of the highly differentiated intestinal epithelial cells, with the same microvilli, cell polarity, and tight connection, which can be utilized to simulate the function of intestinal epithelial cells *in vivo*. Therefore, we chose this cell model to explore the effect of LAB surface proteins on the adhesion characteristics of bacteria. As shown in Figure 3, FITC was used as a marker to track the adhesion of lactic acid bacteria to cells. Lactic acid bacteria with complete surface proteins showed excellent adhesion characteristics. With the removal of the bacterial surface proteins by LiCl, the adhesion ability of lactic acid bacteria to cells also decreased. Flow cytometry data (Figure 4) showed that the adhesion rate of lactic acid bacteria reduced from 98.1 to 20.9%, and LiCl treatment for 3 h had the greatest effect on the adhesion ability of cells. HE staining results showed that *L. plantarum* HC-2 had high adhesion ability to HCT116 cells, and the adhesion rate was decreased significantly after removing the surface proteins of lactobacilli with LiCl (Figure 5).

### Identification of Proteins in the Soluble and Surface Enriched Fractions

**Figure 6:**
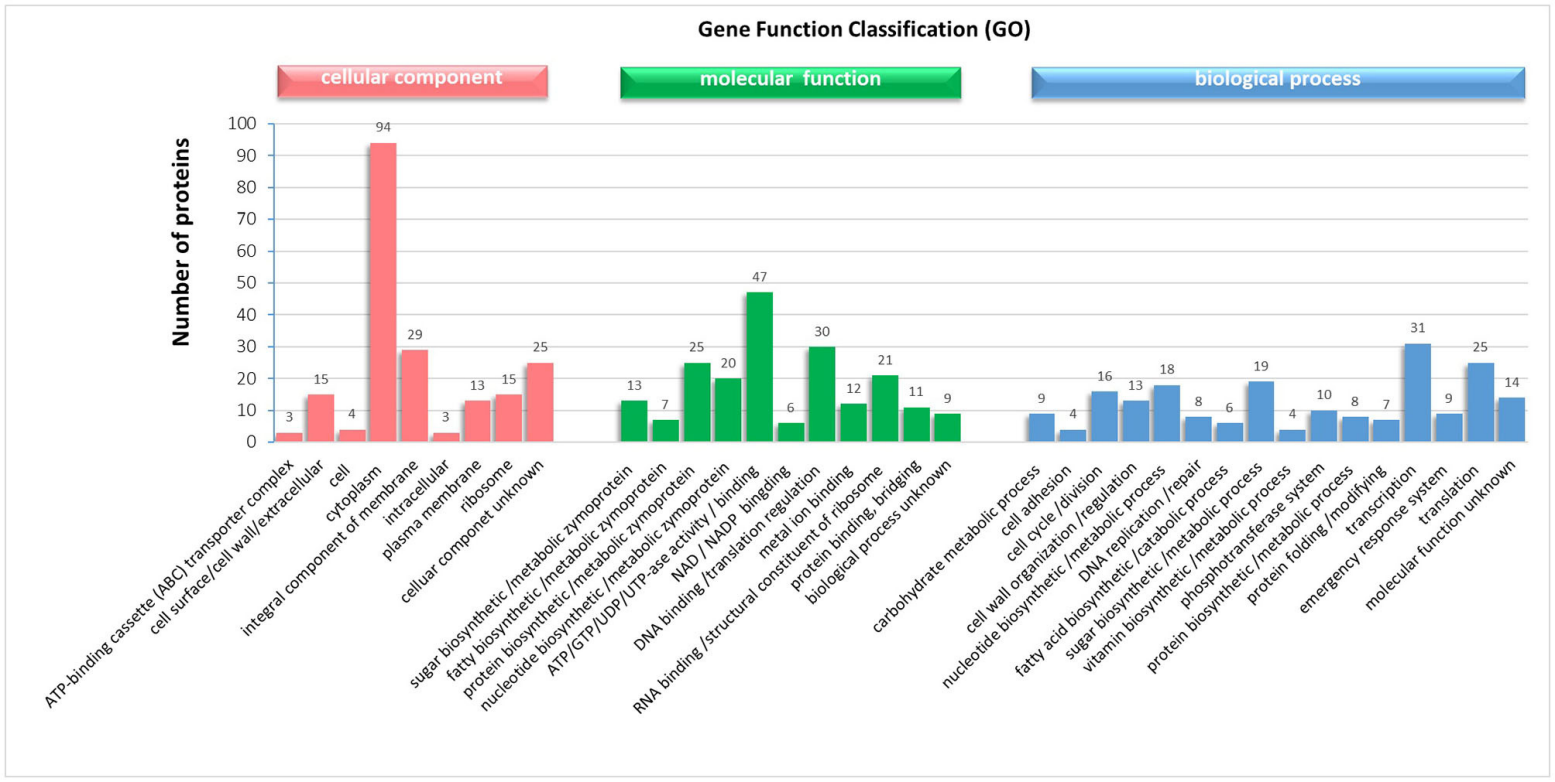
Gene ontology (GO) analysis of the proteins in the mixture proteins obtained from the surface of *L. plantarum* HC-2 with LiCl-treated and identified by LC-MS/MS performance.

From the SDS electrophoresis results, we found that the incubation time of LiCl less than or equal to 9 h significantly increased both the position and content of the bands with longer incubation time, while there was no significant difference with longer incubation time. Also, the level of adhesion of the bacterium to the crude shrimp mucus and collagen decreased significantly with longer incubation time in LiCl, but longer incubation time in LiCl did not reduce the adhesion rate of the bacterium again. Therefore, we collected the surface proteins of the bacteriophage incubated with LiCl for 9 h and identified 201 specific proteins by mass spectrometry (Supplementary Tables S1–S4). Subsequent assessment of the cellular component, biological process, and molecular function of the identified proteins were done based on the Uniprot database (updated July 18, 2018). Meanwhile, using a variety of bioinformatics tools (SignalP, Pfam, LipoP, pSORT, and TMHMM) to verify or to adjust the 176 of 201 protein localizations predicted GO analysis. Cellular component (CC), describing locations, at the levels of subcellular structures and macromolecular complexes, showed that most of the identified proteins were located in the cytoplasm (94), where 29 proteins were an integral component of membrane proteins and enzymes, a small part of the proteins were enriched in the ribosome (15), cell surface/cell wall/extracellular region (15) and plasma membrane (13), respectively, and 25 proteins were not clear (Figure 6). Biological Process (BP) analysis, describing biological goals accomplished by one or more ordered assemblies of molecular functions, showed that the identified proteins were involved in transcription (31), translation (25), sugar biosynthetic or metabolic processes (19), nucleotide biosynthetic or metabolic processes (18), cell cycle or division (16), cell wall organization or regulation (13), phosphotransferase system (10), carbohydrate metabolic process (9), emergency response system (9), protein biosynthetic or metabolic processes (8), DNA replication or repair (8), protein folding or modifying (7), fatty acid biosynthetic or catabolic processes (6), and cell adhesion (4) (Figure 6). Molecular function (MF), describing activities, such as catalytic or binding activities, at the molecular level, showed that the identified proteins perform binding or regulation function for ATP/GTP/UDP/UTP-ase activity (47), DNA translation (30), RNA binding or structural constituent of ribosome (21), metal ion binding (12), protein binding, bridging (11) and NAD/NADP binding (6), or as functional enzyme proteins which participate in protein, nucleotide, and sugar and fatty biosynthetic or metabolic processes (25, 20, 13 and 7, respectively) (Figure 6). In addition, 23 proteins were transmembrane proteins predicted by TMHMM, and 26 proteins have signal peptides predicted by the Signal P (shown in the Supplementary Material).

### The Number of *L. plantarum* HC-2 Colonized in the Intestine of Shrimp

**Figure 7:**
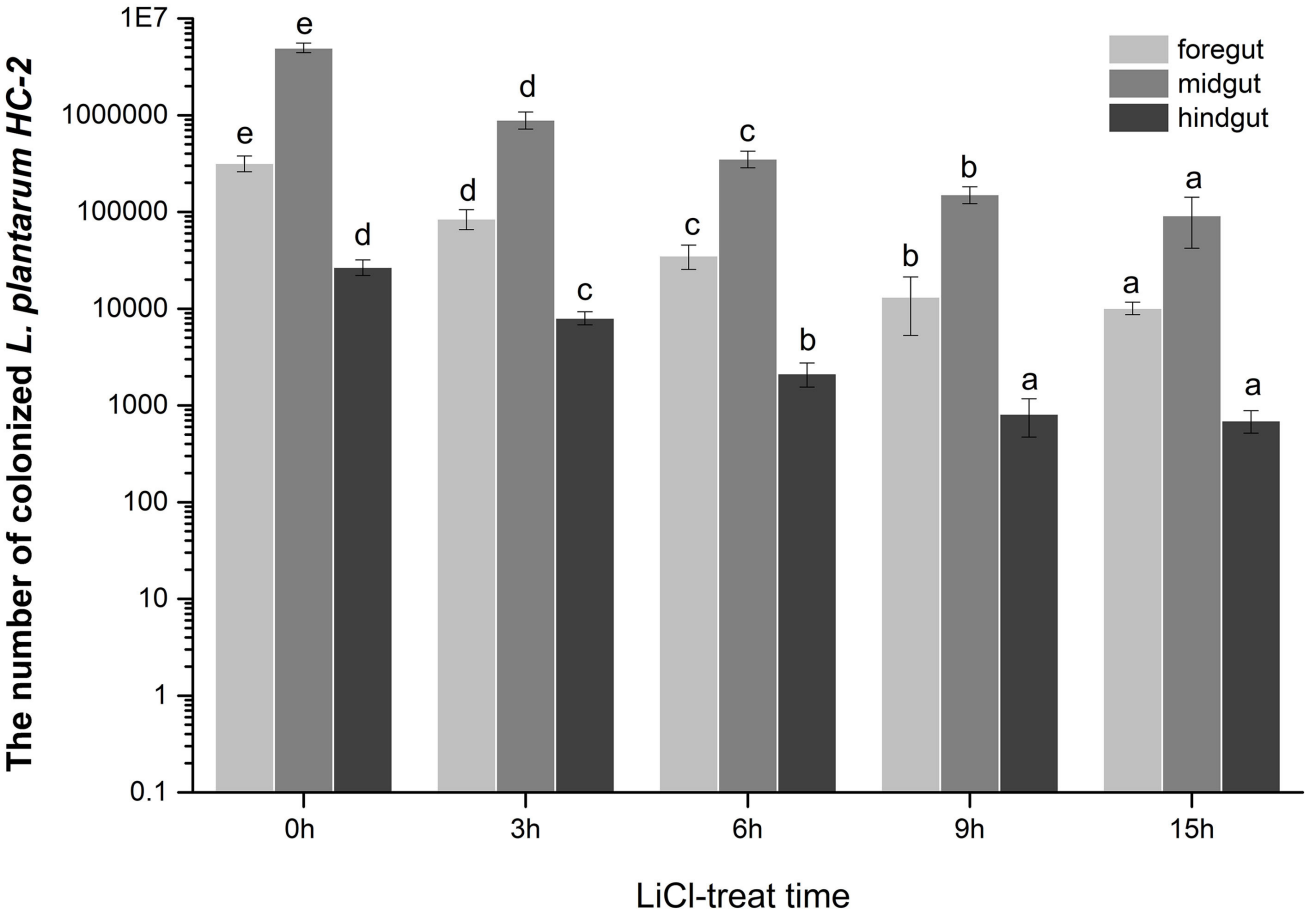
The numbers of the *L. plantarum* HC-2 colonization in the foregut, the midgut, and the hindgut of the shrimp. The bacteria were added to the basal diet (5 × 10^8^ CFU/g feed) and the shrimps were fed continuously for 2 weeks, and the intestine of the shrimp, after one day of starvation was sampled. The intestinal contents were washed with sterile PBS, and then bacterial DNA was extracted from the foregut, midgut, and hindgut respectively, and the number of bacteria was detected by real-time quantitative PCR using a specific primer. Values were means ± SE, and different letters denote significant differences among different groups (*p* < 0.05).

**Figure 8:**
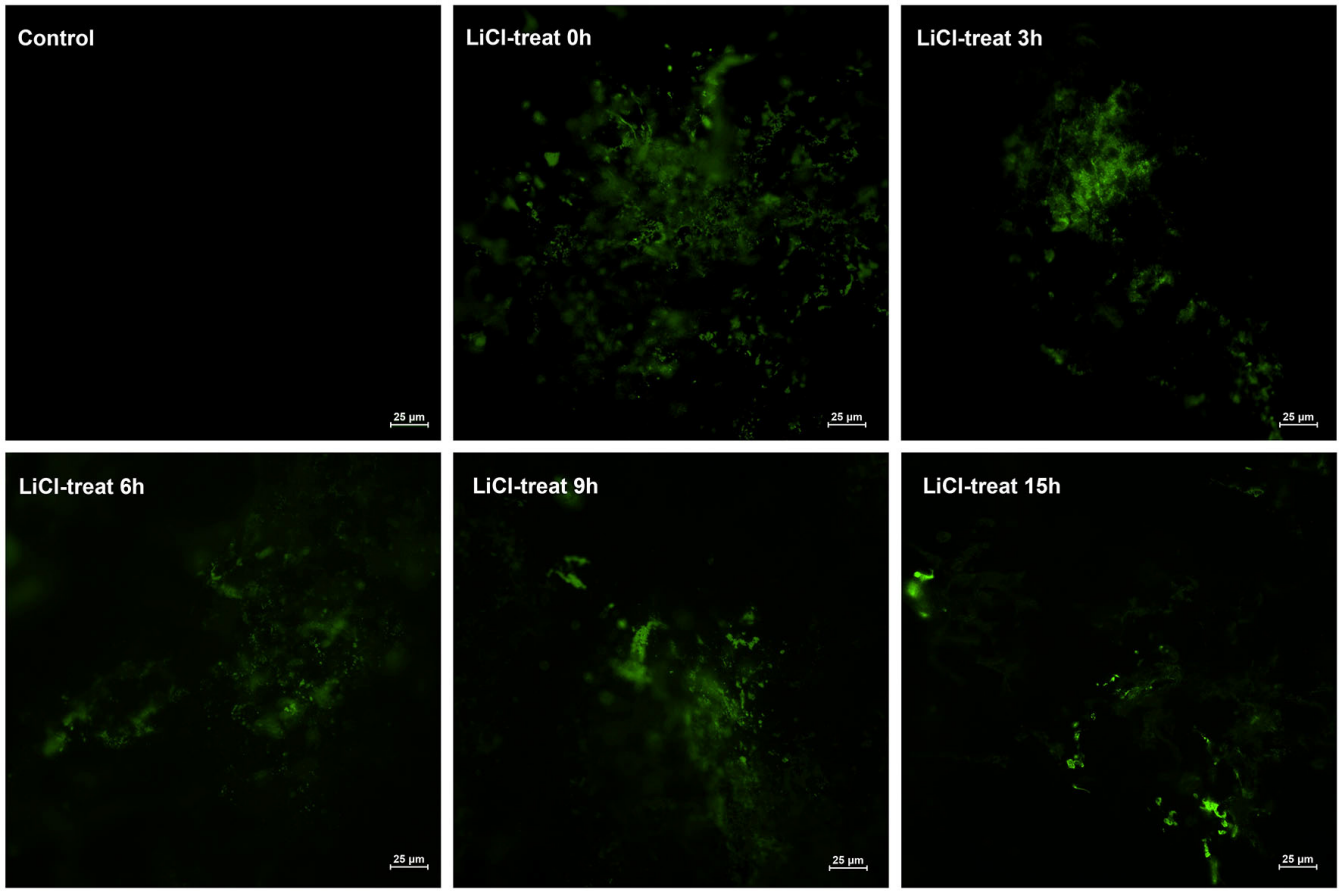
The colonization of *L. plantarum* HC-2 in the foregut of shrimp was observed under the fluorescence microscope. The normal and LiCl-treated bacteria were labeled with FITC and were added to the basal diet (5 × 10^8^ CFU/g feed) to feed shrimps continuously for 2 weeks, and then the foregut was sampled after one day of starvation. The intestinal contents were rinsed with sterile PBS, and the intestinal tract was dissected longitudinally with a sterile scalpel.

Interestingly, we found that lactic acid bacteria colonized mainly in the foregut of shrimp, followed by the midgut. The number of bacteria colonizing in the midgut was approximately 15 times higher than in the foregut, while the number of bacteria colonizing in the hindgut was relatively small. To more accurately explore the effects of surface proteins on LAB colonization, we used qPCR to detect the bacterial colonies in the intestinal tract after the shrimp fed with bacteria were treated by LiCl for different times. The results demonstrate that the colonization rate of normal HC-2 is significantly higher than that in the LiCl-treated bacterial group. The colonization numbers of bacteria in different intestinal segments of shrimp showed a decreasing trend with the extension of LiCl treatment time (Figure 7). Fluorescence microscope observation also confirmed that LiCl treatment could reduce the number of bacterial colonization in the intestine of shrimp (Figure 8). These results noted that surface proteins had a significant effect on the colonization rate of lactic acid bacteria in the intestinal tract of shrimp.

### Adhesive Activity of *L. plantarum* HC-2 to Crude Intestinal Mucus of Shrimp

**Figure 9:**
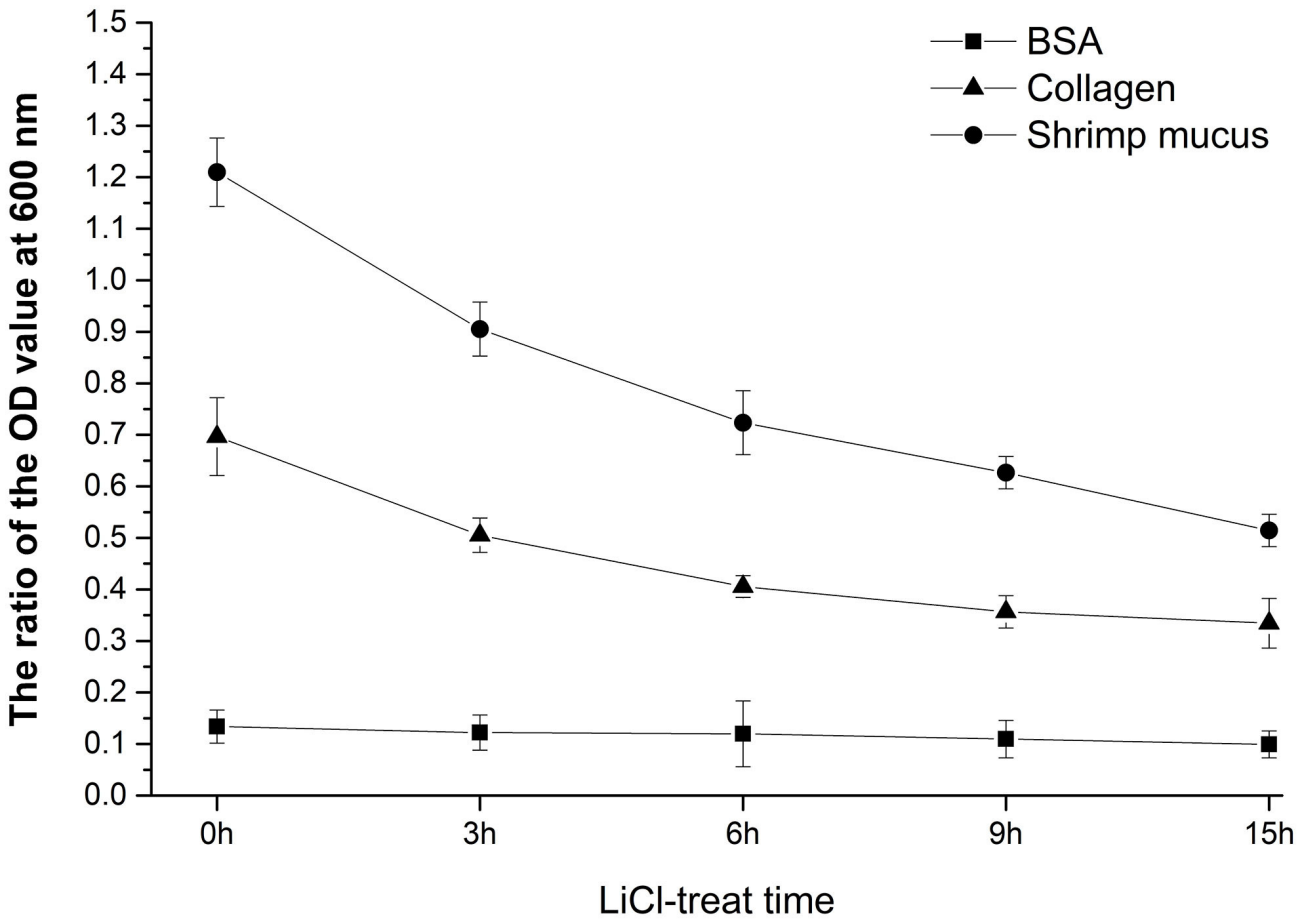
The adhesive rate is presented by the ratio of the fluorescence absorption value at 500 nm of adherent bacteria to initial bacteria. The adhesive activity of LiCl-treated *L. plantarum* HC-2 to bovine serum albumin (BSA), collagen, and crude mucus of *L. vannamei*. Results are mean ± standard deviation for three experiments (three replicates per experiment).

At the optimized labeling condition, the labeling rate of the bacterial cells concentration of 1 × 10^9^ CFU/mL with 20 μmol/L CFDA-SE at 37°C for 20 min could reach at least 95% (data unpublished). Adherence activity studies showed that *L. plantarum* HC-2 could attach to BSA, collagen proteins, and crude intestinal mucus of *L. vannamei* initially, and to crude mucus of shrimp showed the highest adhesive ability (ratio = 1.2099 ± 0.0663) than BSA (ratio = 0.1338 ± 0.0323) and collagen proteins (ratio = 0.6967 ± 0.0756), respectively (Figure 8). The ability of *L. plantarum* HC-2 to adhere to the collagen and crude mucus of shrimp decreased with the increasing time of LiCl treatment. However, the adhesive rate of *L. plantarum* HC-2 to BSA was not decreased with prolonged LiCl-treatment time (Figure 9).

### Identification of Adhesion Proteins on the Surface of HC-2

**Figure 10:**
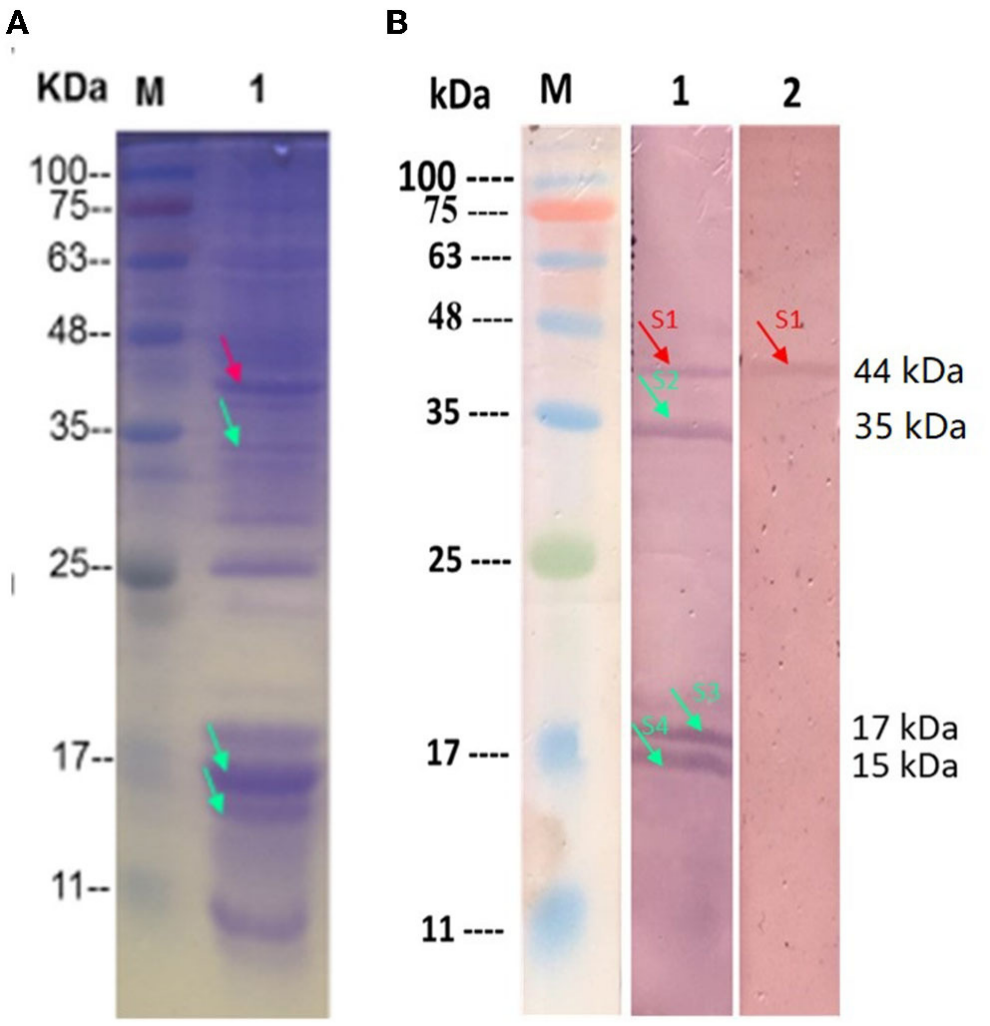
Identification of the surface adhesion proteins in the HC-2. **(A)** The SDS-PAGA electrophoresis results of HC-2 surface protein; **(B)** HC-2 surface adhesion protein By Western blot. M: Marker; Line1: The HC-2 surface protein was transferred to PVDF membrane and were incubated with NHS-shrimp membrane protein; Line2: The HC-2 surface proteins were transferred to PVDF membrane and were incubated with NHS-shrimp mucus protein. Unlabeled shrimp intestinal membrane protein and mucus protein were used as negative controls, and no response bands were detected in WB (data not shown in the picture).

The surface protein of HC-2 was extracted by 5M of neutral LiCl and then transferred to the PVDF membrane. After reacting with NHS-labeled shrimp mid-intestine membrane protein and mucus, four obvious positive bands with molecular weights of about 44 kDa, 36 kDa, 17 kDa, and 15 kDa appear, respectively (Figure 10B). We used MS to identify 44 kDa as Elongation factor Tu, 36 kDa as Type I glyceraldehyde-3-phosphate dehydrogenase, 17 kDa as Small heat shock protein, and 15 kDa as 30S ribosomal protein (Table 1).

**Table 1:** Mass spectrometric identification of HC-2 surface adhesion proteins interacts with the epithelial cells and crude intestinal mucus of shrimp.

**Positive stripe**	**Protein [species]**	**Accession number**	**Predicted MW(kDa)/PI**	**Mascot scores**	**Match peptides**
S1-44 kDa	Elongation factor Tu [*Lactobacillus plantarum*]	A0A3M6KIW5_LACPE	43.392	68	4
S2-35 kDa	Type I glyceraldehyde-3-phosphate dehydrogenase [*Lactobacillus plantarum*]	A0A241RMA5_LACPE	36.416	329	6
S3-17 kDa	Small heat shock protein [*Lactobacillus plantarum*]	*I8R7M0 LACPE*	16.627	5,256.58	15
S4-15 kDa	30S ribosomal protein S8 [*Lactobacillus plantarum*]	*I9ANI7 LACPE*	14.643	700.06	11

## Discussion

The survival of probiotic bacteria in gastrointestinal ecosystems and their adhesion to intestinal mucosa is considered to be preconditions for temporary colonization, stimulation of the immune system, and resistance to intestinal pathogens (Hudault et al., 1997; Roselli et al., 2005). The physiological function of probiotics is closely related to the composition of surface substances, such as phosphoric acid, intact peptides, capsular polysaccharides, and surface proteins, which are directly or indirectly involved in the adhesion and colonization of bacteria. In the present study, we investigated the importance of cell surface proteins in the probiotic activities of *L. plantarum* HC-2 strains including cell surface characteristics, aggregation properties, and adhesion abilities to HCT116 cells and shrimp mucus, and identified the surface proteins by ToF-MS/MS after extracted by 5 M LiCl.

The presence of the external cell surface of *L. plantarum* HC-2 was established by SEM and TEM. Electron photomicrographs showed that the mucus layer was detached after using 5 M LiCl treatment, and the bacterial cells became more decentralized, and the morphology of all three structural layers was changed, especially the outermost layer was missing, together with the electrophoretic analysis showing that the surface proteins can be thoroughly removed using 5 M LiCl treatment. Since the *L. plantarum* HC-2 cells both before and after treatment have biological activities, the adhesion results due to cell death can be excluded, thereby suggesting that surface proteins play an important role in maintaining the cell structure stability and cell adhesion. Studies on the regeneration characteristics of S-layer proteins showed that given proper culture conditions, the treated cells of the bacteria can re-express the surface proteins. The results showed that the basic cellular structure of the cell homeostasis was not damaged. The genetic material was intact, and the normal growth and metabolic activities were not disturbed. This grounding reveals that surface proteins can protect cells from peripheral disturbance. At the same time, it was further confirmed that surface proteins are the biofilm structure of the outer layer of cells. Johnson-Henry et al. (2007) observed *L. helveticus* R0052 bacterial cells by TEM and found that in addition to the cell membrane, cell wall, the outermost layer was packaged by a layer of membrane material, that S-protein, while the intact cell wall structure could still be observed after removing the cell s-layer using LiCl denaturant, which confirms the distribution of S-layer proteins from the microstructure. In *in vitro* adhesion experiment, we first used the HCT116 cell model to study the effect of surface proteins on the adhesion ability of lactic acid bacteria and found that the normal lactic acid bacteria had strong adhesion ability to HCT116 cells, but lactic acid bacteria treated with LiCl could not adhere to HCT116 cells. This suggests that surface proteins are essential for the adhesion properties of lactic acid bacteria.

To better understand the properties of surface proteins from HC-2, the proteins in the extracted mixture were identified by peptide mass fingerprinting and automated MS/MS analysis of major ions in the matrix-assisted laser desorption/ionization (MALDI) ionization mode which can achieve the identifications even when a gel band contains multiple proteins (Mueller et al., 2007). Depending on the local database, 201 proteins were identified. According to GO-cellular component classification, the proteins were mainly classified into cytoplasmic proteins, secreted proteins, and surface-associated proteins. GO bioprocess annotation results indicate that proteins localized to the cytoplasm account for 48.26% of the total identified proteins and that these proteins may require some other modifications to anchor to the cell surface in addition to using specific binding motifs or structural domains to bind to the cytoplasmic membrane, which requires more in-depth structural analysis and localization prediction. Many of the identified cytoplasmic proteins are highly abundant proteins, such as cell cycle or cytokinesis proteins (as trigger factor, cell cycle protein GpsB, nucleoid occlusion protein, cell division protein FtsZ, etc.), transcription and translation (as MarR family transcriptional regulator, Phenylalanine-tRNA ligase beta subunit, Ribosome-recycling factor, RNase P protein, etc.), enzymes involved in glucose metabolic process (as glucose-6-phosphate isomerase, glyceraldehyde 3-phosphate dehydrogenase, glucokinase, L-lactate dehydrogenase, etc.), and emergency and modified proteins (as Protein GrpE, Protein Cpn60, Hsp20). Although many of the intracytoplasmic proteins identified in the study are readily released during cell lysis causing some degree of contamination, rather than causing the secretion of the active material outside the cell, it has also been speculated that certain cytoplasmic proteins may have extracellular functions. One example is EF-Tu which has been proved to be a vital mucin adhesion factor identified at the surface of several Lactobacillus species (Mukai et al., 2002; Granato et al., 2004; Dhanani and Bagchi, 2013; Nishiyama et al., 2013). Except for EF-Tu, we also identified EF-G, EF-P, and EF-Ts proteins and this implies an exposed localization, similar to the report in E. faecalis V583 (Bøhle et al., 2011) and model B. subtilis (Tjalsma et al., 2008). In bacteria, EF-G catalyzes the GTP-dependent ribosomal translocation step during translation elongation; EF-P is involved in peptide bond synthesis, and EF-Ts is associated with the EF-Tu-GDP complex and induces the exchange of GDP to GTP. Extracellular DnaK, enolase, and GAPDH were identified in this study and in the laboratory strain, E. faecalis JH2-2 (Benachour et al., 2009), these proteins may play an important role in bacterial adhesion, as they have been reported to have a strong binding capacity to human thrombospondin (Schaumburg et al., 2004).

The assembly of prokaryotic ribosomal subunits takes place in the nucleolus and requires the nuclear import of ribosomal proteins. In the present study, the subcellular location of 16 cytoplasmic proteins was predicted on ribosomal proteins according to GO annotation, including 30S ribosomal proteins (S2, S3, S5, S7, and S9), 50S ribosomal proteins (L2, L3, L4, L5, L11, L13, L15, L23, and L7/L12), and a ribosome hibernation promoting factor (HPF). Recently, ribosomal proteins have been identified not only in the cytoplasm but also on the surface of bacteria, and they may play a key role in mediating bacterial adhesion and colonization; for example, surface-associated ribosomal proteins have been detected in *Streptococcus pseudomallei* and *Bacillus subtilis* 168 (Schaumburg et al., 2004; Severin et al., 2007; Tjalsma et al., 2008; Benachour et al., 2009). In addition, high levels of ribosomal proteins on the surface of bacteria are highly immunogenic in many immune processes and can effectively induce an immune response in the organism (Kurar and Splitter, 1997; Spence and Clark, 2000), making them important targets for current study on vaccine candidate antigens, such as Rbp L7/L12, which has been found on the surface of several Gram-positive bacteria (Rodríguez-Ortega et al., 2006; Severin et al., 2007; Tjalsma et al., 2008). However, whether these proteins are keeping their biological activity at the bacterial surface, or do “moonlighting”, i.e., acquiring another function when surface-exposed, remains still unclear (Jeffery, 2003, 2014, 2016, 2019). So, the determination of the exact number of ribosomal proteins is important for the precise knowledge about the structure and function of ribosomes.

Integral membrane proteins participate in a variety of activities essential for survival, homeostasis, and division of the cell. Many functions only within the dynamic multi-protein assemblage embedded in specialized lipid microdomains of the bacterial cytoplasmic membrane (CM). In this work, 14.93% of the total identified proteins were an integral component of membrane proteins predicted by GO annotation, but the subcellular location for these proteins might not be unclear. Among them, six proteins (Penicillin-binding protein, glycoside hydrolase family 25, transpeptidase-transglycosylase, LysM domain-containing protein, extracellular transglycosylase, and D-alanyl-D-alanine carboxypeptidase) were mainly involved in cell wall macromolecule catabolic and peptidoglycan catabolic processes. Penicillin-binding proteins (PBPs) are a group of proteins present on the surface of certain bacteria, and are one of the common structures of bacteria, which can bind specifically to penicillin and make penicillin completely antigenic, and are essential proteins in the process of cell division, catalyzing the cross-linking of peptidoglycan cell walls in the division compartment (King et al., 2017). Transpeptidases are enzymes that recognize specific polypeptides during protein biosynthesis and catalyze different transpeptidase reactions. The enzymes contain a penicillin-insensitive transglycosylase structural domain at the N-terminus and a penicillin-sensitive transpeptidase structural domain at the C-terminus, which is anchored to the cell surface by transmembrane structures and participate in the formation of the bacterial cell wall (King et al., 2017).

The cell wall of Gram-positive bacteria was covered by a thick and multilayered Peptidoglycan layer which is decorated with lipoteichoic acid (LTA), wall teichoic acid (WTA), and various proteins, including S-layer proteins and membrane proteins. The S-layer proteins are attached to the cell wall through charged or uncharged secondary cell wall polymers, whereas membrane proteins can covalently attach to the long-chain fatty acids of the cytoplasmic membrane. Cell wall-anchored proteins are attached to the cell *via* their C-terminus, either (i) covalently by sortases (e.g., LPXTG proteins) or (ii) non-covalently [e.g., through a LysM motif or cell wall-binding domains (CWBDs)]. Based on the GO classification, 15 identified proteins were predicted location on the cell surface or cell wall. A protein named cell surface protein was identified with a cell wall anchor motif of LPQTN. SAAS annotation, to predict its subcellular location as a cell wall or peptidoglycan-anchor, has a signal peptide and a collagen bind domain, mainly displaying cell adhesion. Mucus-binding protein has a signal peptide and ten MucBP (MUCin-Binding Protein) domain which is found in a wide variety of bacterial proteins, in several reports (Singh et al., 2018; Xiong et al., 2018). The domain is found in bacterial peptidoglycan bound proteins and is often found in conjunction with LPQTD cell wall anchor motifs and Leucine-rich repeat motifs. Lipoproteins may be involved in the regulation of cell wall degradation during bacterial isolation or cell division, and negatively regulate the production of extracellular DNA (eDNA) (Ohara et al., 1999; Sanchez-Torres et al., 2010). Chaperone protein DnaK (HSP70) anchored on the cell surface by membrane-bound, usually found on the surface of bacteria (Saad et al., 2009), plays an important role in the initiation of phage lambda DNA replication, possibly through similar interactions with the DnaA protein, and is also actively involved in the response to hyperosmotic shock. The cell-surface architecture (integral membrane) of Gram-positive bacteria consisted of the cytoplasmic membrane, peptidoglycan layer, and S-layer (Nishiyama 2013). In the present study, 5.47% of the total identified proteins were predicted to be located in the plasma membrane. Interestingly, there was no transmembrane domain prediction found in these proteins, but most proteins were identified as having signal peptide and SPaseI-cleavage sites, which indicates that most plasma membrane proteins isolated by LiCl were secretory proteins. In gram-positive bacteria, the solute-binding proteins are membrane-anchored lipoproteins; thus, the bacterial high-affinity transport systems play an essential role in the active transport of solutes across the cytoplasmic membrane.

The foregut of shrimp includes the mouth, esophagus, and stomach, which mainly digest food, while the midgut has microvillous structures that mainly perform absorption functions, and the hindgut includes the rectum and anus, which mainly exercise excretion (Martin et al., 2010). We found that HC-2 mainly colonized the midgut of shrimp, with 15 times more colonization than the foregut. This may be related to the fact that HC-2 has abundant surface proteins that can interact with the epithelial cells of the midgut. Several studies have shown that the surface proteins of bifidobacterial (Guglielmetti et al., 2008) and lactobacilli (Johnson-Henry et al., 2007), even the pathogenic bacteria (Engelhardt, 2007) mediate or/and promote the adhesion of bacteria to the intestinal mucus and epithelial cells. Next, we used crude intestinal mucus of *L. vannamei* as an adhesion model. It was also found that HC-2 had significantly higher adhesion to mucus than to BSA, a non-specific adhesion control. And adhesion to mucus was decreased significantly with the prolonged LiCl-treatment time but was still higher than to BSA. In the beginning, the mucus layer coated on HC-2 contributed to shrimp crude mucus and BSA adhesion, and the adhesion was decreased with the mucus layer breaking off the surface of the bacteria. Thereafter, the detachment of surface proteins results in the decrease of adhesion. The result also supports the conclusion that numerous factors are involved in LAB adhesion. It is necessary to demonstrate that the adhesion of HC-2 to mucus is mediated by bacterial surface-specific proteins. Collagen is commonly used to isolate and characterize the collagen-binding S-layer proteins in bacteria (Toba et al., 1995). Our results also revealed that HC-2 specifically adheres to collagen, but at a lower rate compared to the crude mucus of shrimp. This may be due to the fact that collagen is a single component and it has fewer interactions with bacterial surface proteins, whereas there are multiple proteins in mucus that can interact with bacterial surface proteins, but both interactions cannot fully explain the actual adhesion mechanism of lactic acid bacteria in the organism. The adhesion characteristics were common to many S-layer proteins (Strus et al., 2001; Ying et al., 2007). Furthermore, we identified that the surface proteins of Elongation factor Tu, Type I glyceraldehyde-3-phosphate dehydrogenase, Small heat shock protein, and 30S ribosomal are possible to interact with the shrimp intestinal proteins by Western blotting. Among them, EF-Tu and GAPDH were reported to be involved in mediating bacterial adhesion (Zhang et al., 2016; Wang et al., 2018), but their mediation of the interaction between bacteria and shrimp intestinal protein has not been studied. Otherwise, there must be some differences in the adhesion characteristics of the bacterial surface proteins of different species, especially when the coexistence of probiotics and pathogenic bacteria all can express surface proteins. What are the competing mechanisms for the adhesion of pathogenic and probiotic bacteria to intestinal epithelial cells and mucus, and these questions will be investigated in depth at the genetic level. Therefore, we will perform a subsequent further study on the interaction mechanism of the adhesion protein and the receptor protein. This will be helpful to explore the mechanism of the colonization of lactic acid bacteria in the intestine, the signal exchange between lactic acid bacteria and the intestinal cells of shrimp, and the inhibition of the growth of pathogenic bacteria in the intestine by lactic acid bacteria, which is of great significance for the improvement of the microecological environment of the digestive tract and the ecological control of the pathogenic bacteria.

## Conclusion

In conclusion, our studies reveal that the surface proteins of *L. plantarum* HC-2 play a vital role in maintaining stable cell morphology and mediating cell adhesion. The adhesive ability of *L. plantarum* HC-2 to the HCT116 cells and the crude intestinal mucus of shrimp was dependent on the surface proteins. The surface proteins include Elongation factor Tu, Type I glyceraldehyde-3-phosphate dehydrogenase, Small heat shock protein, and 30S ribosomal proteins are possible adhesion proteins, which may mediate the bacteria colonization. In our future study, we will identify the receptor proteins of these proteins in the intestinal tract of shrimp and study the function of their interaction in regulating the intestinal immunity of shrimp. This study will provide the theoretical foundation and application promotion of surface proteins in LAB and therefore might help us further understand the mechanisms of adhesion/colonization and probiotic function of *L. plantarum* HC-2 in the intestines of shrimp.

## Data Availability Statement

The original contributions presented in the study are included in the article/Supplementary Material, further inquiries can be directed to the corresponding author.

## Author Contributions

YD and JC designed the experiments and YD wrote the manuscript. HL, TW, WX, and XH performed all of the experiments. JS and JC gave support and advice on the experiments. All authors have read and agreed to the published version of the manuscript.

## Funding

This work was supported by the National Natural Science Foundation of China (41906107), the Key Scientific and Technological Grant of Zhejiang for Breeding New Agricultural Varieties (2021C02069-5), and the fund from the Key Laboratory of Marine Biotechnology of Fujian Province (2021MB04).

## Conflict of Interest

The authors declare that the research was conducted in the absence of any commercial or financial relationships that could be construed as a potential conflict of interest.

## Publisher's Note

All claims expressed in this article are solely those of the authors and do not necessarily represent those of their affiliated organizations, or those of the publisher, the editors and the reviewers. Any product that may be evaluated in this article, or claim that may be made by its manufacturer, is not guaranteed or endorsed by the publisher.
