# Supplementary material for: Adhesion and Colonization of the Probiotic Lactobacillus plantarum HC-2 in the Intestine of Litopenaeus Vannamei Are Associated With Bacterial Surface Proteins
Source: Front Microbiol. 2022 Apr 13;13:878874. doi: 10.3389/fmicb.2022.878874 (PMC9076606; doi:10.3389/fmicb.2022.878874)
Supplement: Supplementary file 1 [file Data_Sheet_1.PDF]

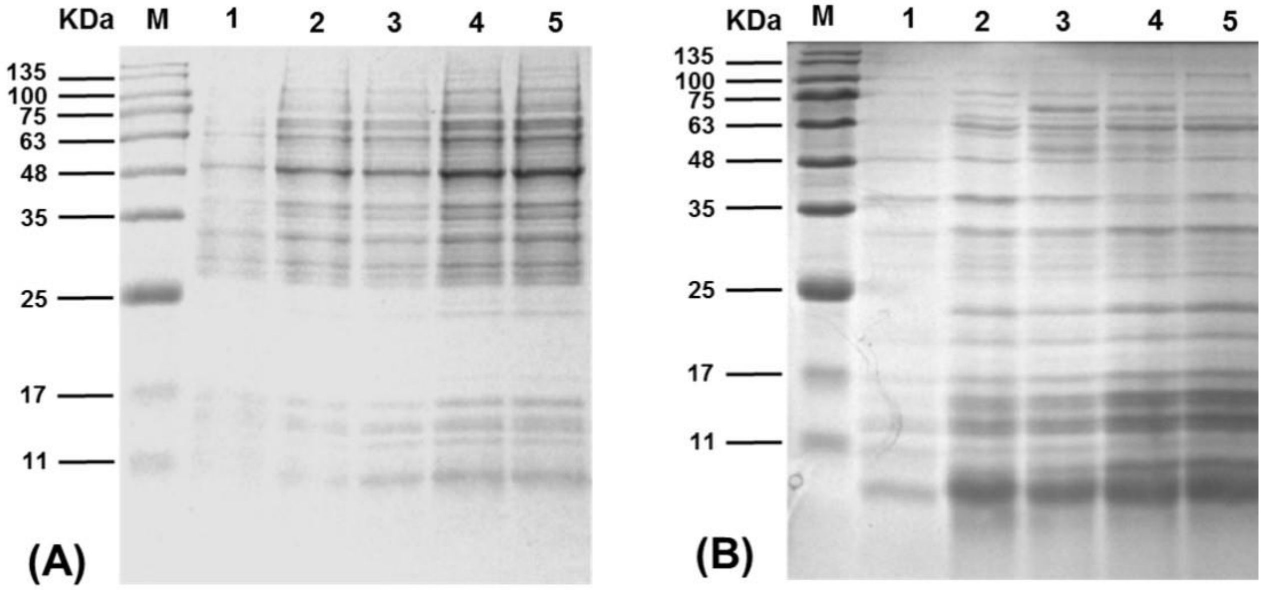

Fig. S1. The whole bacteria protein and the surface-associated protein were analyzed by SDS-PAGE (15% separating gel, at 80 V for 150 min). Distinct protein components were shown in the gel after staining with Coomassie brilliant blue R-250. **A:** the bacteria were treated with LiCl for different times (lane M: marker; lane 1-5: LiCl treat for 0, 3, 6, 9, and 15 hours). **B**: the cell surface proteins extract with LiCl-treatment (lane M: marker; lane 1-4: LiCl treat for 3, 6, 9, and 15 hours; lane 5: the regeneration of cell surface proteins from the LiCl-treated bacteria).

Table 1. Surface-associated proteins of *Lactobacillus. plantarum* HC-2 located in cytoplasm were identified by LC-MS/MS.

Accession [a]	Protein names	Sum PEP Score [b]	Coverage	Area [c]	PSMs	Unique Peptides	MW [d] [kDa]	calc. pI [e]	Gene ontology (biological process)	Gene ontology (cellular component)	Gene ontology (molecular function)
A0A241RKR1	Translation initiation factor IF-3	132.46	58.96	1.68E+10	70	12	19.7	9.95	formation of translation preinitiation complex; positive regulation of formation of translation preinitiation complex; ribosome disassembly; translational initiation	cytoplasm	translation initiation factor activity
A0A241RQB7	Heat-shock protein Hsp20	157.76	53.74	3.41E+10	234	14	16.6	5.15	protein stabilization; response to heat; stress response to copper ion	cytoplasm	identical protein binding
A0A241RQF9	Transcriptional repressor	23.13	18.13	3.39E+8	6	3	18.1	6.43	regulation of transcription, DNA-templated; response to stress; transcription, DNA-templated	cytoplasm	DNA binding; DNA-binding transcription factor activity
A0A241RQJ4 [f, g]	D-alanyl-D-alanine carboxypeptidase	71.70	23.87	7.36E+9	14	8	46.9	9.99	ell wall organization; peptidoglycan biosynthetic process; regulation of cell shape; response to drug	cytoplasm	serine-type D-Ala-D-Ala carboxypeptidase activity
A0A241RQP6	Chromosome partitioning protein ParB (ParB/ RepB/Spo0J family partition	88.43	48.65	1.71E+9	38	15	33.2	9.07	chromosome segregation	cytoplasm	sequence-specific DNA binding
A0A241RSL8	Site-determining protein	16.27	13.43	1.27E+8	3	3	29.1	5.21	cell division;cell morphogenesis; cell septum assembly; regulation of cell septum assembly	cytoplasm	ATPase activity; ATP binding
A0A241RSP2	Probable GTP-binding protein EngB	16.13	19.80	1.80E+8	3	3	22.4	8.92	cell septum assembly	cytoplasm	GTP binding ; metal ion binding
A0A2I0Z5Y8	RNA-binding protein S1	66.16	37.22	2.62E+9	17	6	19.4	9.48	RNA modification	cytoplasm	nucleic acid binding
A0A2I0Z6E6	Nucleoid occlusion protein	27.38	25.80	1.10E+8	11	1	31.7	9.31	cell cycle; cell division; negative regulation of division septum assembly	cytoplasm	DNA binding; DNA-directed 5'-3' RNA polymerase activity; protein dimerization activity
A0A2K9HY48	Aldo/keto reductase	21.60	12.77	2.99E+8	7	5	36.7	6.46	oxidation-reduction process	cytoplasm	oxidoreductase activity
A0A2K9I3F9	Dipeptidase PepV	26.71	16.92	9.87E+7	6	5	50.8	4.67	peptide catabolic process; peptide metabolic process; proteolysis	cytoplasm	dipeptidase activity ; zinc ion binding
A0A2K9I4M5	MarR family transcriptional regulator	89.94	40.61	1.19E+9	20	8	19.5	6.3	transcription, DNA-templated	cytoplasm	DNA binding ; DNA-binding transcription factor activity
A0A2K9IAM3	Beta-ketoacyl-ACP reductase	23.52	23.53	6.50E+7	4	4	27.4	9.54	poly-hydroxybutyrate biosynthetic process	cytoplasm	oxidoreductase activity
A0A2S9WAK0	Ribosome-binding ATPase YchF	60.36	35.79	2.15E+8	11	10	39.9	4.83	response to oxidative stress	cytoplasm	ATPase activity; ATP binding; GTP binding; ribosomal large subunit binding
F6IYI3	Metal-dependent regulator	35.28	24.19	4.54E+8	11	5	23.8	5.82	transcription, DNA-templated	cytoplasm	DNA binding ; DNA-binding transcription factor activity ; protein dimerization activity ; transition metal ion binding
G0LYZ7	Cys-tRNA(Pro)/Cys-tRNA(Cys) deacylase	51.92	38.92	5.49E+8	9	6	18.6	8.91	translation	cytoplasm	aminoacyl-tRNA editing activity; lyase activity
G0M010	Transcriptional regulator GlnR	17.13	31.03	3.98E+8	6	4	16.9	9.55	regulation of transcription, DNA-templated	cytoplasm	DNA binding
G0M2S9	Glyceraldehyde 3-phosphate dehydrogenase	285.67	74.12	4.53E+8	98	1	36.4	5.54	glucose metabolic process	cytoplasm	NAD binding; NADP binding
I8R9B7	Cysteine desulfurase associated protein, DUF1831 family	22.63	46.90	8.03E+8	6	5	12.5	8.38	cluster assembly	cytoplasm	2 iron, 2 sulfur cluster binding; cysteine desulfurase activity; metal ion binding; pyridoxal phosphate
I8RAE0	Nicotinate-nucleotide adenylyltransferase	15.09	23.70	3.98E+7	3	3	24.1	6.68	NAD biosynthetic process	cytoplasm	ATP binding ; nicotinate-nucleotide adenylyltransferase activity
I8RBV9	Nucleotide-binding protein	12.64	13.61	1.41E+8	3	3	33.3	5.38	apoptotic process; cell cycle; cellular response to growth factor stimulus; gastrulation; negative regulation of cell growth	cytoplasm	ATP binding ; GTP binding
I9AP72	NAD-dependent glyceraldehyde-3-phosphate dehydrogenase	274.32	74.12	6.07E+8	102	1	36.4	5.43	glucose metabolic process	cytoplasm	NAD binding; NADP binding
I9KXE3	Ribonuclease P protein component (RNase P protein)	26.35	39.47	8.57E+8	8	6	13.2	10.32	tRNA 5'-leader removal	cytoplasm	ribonuclease P activity; tRNA binding
I9L2I5	DNA-directed RNA polymerase subunit alpha (RNAP subunit alpha)	164.06	42.04	4.22E+9	42	17	34.8	4.92	transcription, DNA-templated	cytoplasm	DNA binding; DNA-directed 5'-3' RNA polymerase activity; protein dimerization activity
A0A241RKQ2	Arginine repressor	29.79	27.63	6.16E+8	10	4	17.4	8.87	arginine biosynthetic process; protein complex oligomerization; transcription, DNA-templated	cytoplasm	arginine binding; DNA binding; DNA-binding transcription factor activity
A0A241RL72	S-adenosylmethionine synthase	35.35	21.52	3.55E+8	6	6	42.6	5.03	one-carbon metabolic process; S-adenosylmethionine biosynthetic process	cytoplasm	ATP binding; magnesium ion binding; methionine adenosyltransferase activity
A0A241RSS2	Adenine phosphoribo-syltransferase (APRT)	21.57	32.56	2.08E+8	4	4	18.7	5.38	adenine salvage; AMP salvage; purine ribonucleoside salvage	cytoplasm	adenine phosphoribosyltransferase activity
A0A241RTM0	4-hydroxy-tetrahydrodipicolinate reductase	19.34	19.92	4.60E+8	7	1	28.6	5.19	diaminopimelate biosynthetic process; lysine biosynthetic process via diaminopimelate	cytoplasm	4-hydroxy-tetrahydrodipicolinate reductase; NAD binding; oxidoreductase activity, acting on CH or CH2 groups, NAD
A0A2I0Z3U3	3-hydroxyacyl-[acyl-carrier -protein] dehydratase FabZ	53.14	37.41	9.91E+8	19	7	16	8.44	fatty acid biosynthetic process; lipid A biosynthetic process	cytoplasm	3-hydroxyoctanoyl-[acyl-carrier-protein] dehydratase activity
A0A2I0Z8A4	Glutamate--tRNA ligase	97.34	32.26	8.06E+8	23	15	57.1	6.57	glutamyl-tRNA aminoacylation	cytoplasm	ATP binding; glutamate-tRNA ligase activity; tRNA binding; zinc ion binding
A0A2K9I473	Hypoxanthine phosphoribosyltransferase	16.18	20.00	9.50E+7	3	3	20.2	5.17	IMP salvage; purine ribonucleoside salvage	cytoplasm	guanine/hypoxanthine phosphoribosyltransferase activity; metal ion binding; nucleotide binding
A0A2S9W0F0	Manganese-dependent inorganic pyrophosphatase	35.68	13.92	9.08E+8	7	4	33.4	4.63		cytoplasm	pyrophosphatase activity
F6IRP1	ATP-dependent 6-phosphofructokinase	79.31	50.00	3.96E+8	16	11	34.2	5.5	fructose 6-phosphate metabolic process	cytoplasm	6-phosphofructokinase activity; ATP binding; metal ion binding
F6ISE1	Glucokinase	33.35	11.96	1.94E+8	5	4	34.5	5.21	glycolytic process	cytoplasm	glucokinase activity
F6IXH7	Lysine--tRNA ligase	20.63	12.42	1.05E+8	6	6	57.5	5.22	lysyl-tRNA aminoacylation	cytoplasm	ATP binding; lysine-tRNA ligase activity; magnesium ion binding; nucleic acid binding
F6IZ25	Phosphoenolpyruvate-protein phosphotransferase	40.91	14.41	4.58E+8	10	7	63.2	4.82	phosphoenolpyruvate-dependent sugar phosphotransferase system	cytoplasm	kinase activity; metal ion binding; phosphoenolpyruvate-protein phosphotransferase activity
F6IZK3	Triosephosphate isomerase (TIM) (TPI)	175.59	70.63	2.67E+9	30	16	27	4.74	gluconeogenesis; glycolytic process	cytoplasm	triose-phosphate isomerase activity
F6IZK4	Phosphoglycerate kinase	327.88	79.75	4.63E+9	99	28	42.8	5.2	glycolytic process	cytoplasm	ATP binding; phosphoglycerate kinase activity
G0M158	Probable transcriptional regulatory protein	203.19	47.93	8.01E+9	56	10	26.4	4.87	regulation of transcription, DNA-templated;	cytoplasm	DNA binding
I8R9P7	Chaperone protein DnaJ	127.48	43.95	1.77E+9	42	16	40.5	8.24	DNA replication; protein folding; response to heat	cytoplasm	ATP binding; heat shock protein binding; unfolded protein binding; zinc ion binding
I9AKF4	Peptide chain release factor 1 (RF-1)	33.92	25.28	2.36E+8	8	8	41	5.02	translational termination	cytoplasm	translation release factor activity, codon specific
I9L008	Ribosome-recycling factor (RRF) (Ribosome-releasing factor)	169.35	73.80	6.25E+9	58	15	20.6	6.11	translational termination	cytoplasm	ribosomal large subunit binding
I9L053	Trigger factor (TF)	455.94	76.35	2.13E+10	217	35	49.9	4.63	cell cycle; cell division; protein folding; protein transport	cytoplasm	peptidyl-prolyl cis-trans isomerase activity

I9L355	60 kDa chaperonin (GroEL protein) (Protein Cpn60)	698.88	63.77	5.54E+10	712	49	57.4	4.78	protein refolding	cytoplasm	ATP binding [GO:0005524]; unfolded protein binding
A0A241RKU0	Phenylalanine--tRNA ligase beta subunit	7.88	8.92	6.61E+7	1	1	22.9	4.63	translation	cytoplasm	ATP binding; metal ion binding; phenylalanine-tRNA ligase activity; tRNA binding
A0A241RLF2	Guanylate kinase	42.85	26.21	4.65E+8	7	5	23.5	5.1	'de novo' pyrimidine nucleobase biosynthetic process; 'de novo' UMP biosynthetic process	cytoplasm	ATP binding; guanylate kinase activity
A0A241RMC0	SsrA-binding protein	33.64	32.05	5.24E+8	10	5	18.1	10.2	trans-translation	cytoplasm	RNA binding
A0A241RN61	L-lactate dehydrogenase (L-LDH)	135.24	40.94	4.50E+9	50	17	34.2	5.05	glycolytic process	cytoplasm	L-lactate dehydrogenase activity
A0A241RSN4	DEAD-box ATP-dependent RNA helicase CshB	23.88	14.99	1.08E+8	7	7	50.6	9.85	response to cold; RNA catabolic process	cytoplasm	ATP binding; ATP-dependent RNA helicase activity; RNA binding
A0A241RTF0	RNA polymerase sigma factor SigA	166.84	56.52	8.79E+8	30	17	41.4	5.57	transcription initiation from bacterial-type RNA polymerase promoter	cytoplasm	DNA binding; DNA-binding transcription factor activity; sigma factor activity
A0A2I0Z043	Serine hydroxymethyltransferase	88.78	32.77	4.15E+8	13	9	44.4	5.67	glycine biosynthetic process from serine; tetrahydrofolate interconversion	cytoplasm	glycine hydroxymethyltransferase activity; methyltransferase activity; pyridoxal phosphate binding
A0A2I0Z126	Ribokinase (RK)	18.06	15.31	3.15E+8	6	4	31.9	4.84	D-ribose catabolic process	cytoplasm	ATP binding; metal ion binding; ribokinase activity
A0A2I0Z1S1	Regulatory protein RecX	10.56	3.75	1.22E+8	2	1	31.1	9.06	regulation of DNA repair	cytoplasm	enzyme binding ATP binding; damaged DNA binding; DNA-dependent ATPase activity; single-stranded DNA binding
A0A2K9I041	D-alanine--D-alanine ligase	42.66	30.54	1.58E+8	6	6	41.3	4.84	cell wall organization; peptidoglycan biosynthetic process; regulation of cell shape	cytoplasm	ATP binding; D-alanine-D-alanine ligase activity; metal ion binding
A0A2K9I3N4	Protein RecA (Recombinase A)	44.14	27.78	5.90E+8	13	8	40.6	5.88	DNA recombination; DNA repair; SOS response	cytoplasm	ATP binding; damaged DNA binding; DNA-dependent ATPase activity
A0A2K9I9T4	Glutamine--fructose-6-phosphate aminotransferase	28.15	10.91	1.18E+8	6	5	65.7	5.21	carbohydrate derivative biosynthetic process; carbohydrate metabolic process	cytoplasm	carbohydrate derivative binding; glutamine-fructose-6-phosphate transaminase activity
A0A2S9VS35	Elongation factor Ts (EF-Ts)	194.72	42.81	1.26E+10	97	15	31.6	5.1	mitochondrial translational elongation; regulation of DNA-templated transcription, elongation; regulation of mitochondrial translation; translational elongation	cytoplasm	translation elongation factor activity
A0A2S9W4L4	UDP-N-acetylmuramoyl-L-alanyl-D-glutamate--2,6-diaminopimelate ligase	42.36	20.69	2.82E+8	7	7	53.8	5.96	cell division; peptidoglycan biosynthetic process; regulation of cell shape	cytoplasm	ATP binding; magnesium ion binding
A0A2S9W748	Redox-sensing transcriptional repressor Rex	24.27	26.67	2.74E+8	6	6	25.3	5.2	negative regulation of transcription, DNA-templated; response to redox state	cytoplasm	coenzyme binding; DNA binding; DNA-binding transcription factor activity
A0A2S9WAA4	Glucose-6-phosphate isomerase (GPI)	218.41	41.11	2.95E+9	49	18	49.8	5.08	gluconeogenesis; glycolytic process	cytoplasm	glucose-6-phosphate isomerase activity
F6IRF3	Protein GrpE (HSP-70 cofactor)	116.91	41.90	2.24E+9	36	11	22.8	5.24	protein folding	cytoplasm	adenyl-nucleotide exchange factor activity; chaperone binding; protein homodimerization activity
F6IVR2	10 kDa chaperonin (GroES protein) (Protein Cpn10)	84.93	87.23	3.92E+10	140	10	10.3	4.92	protein folding	cytoplasm	ATP binding
F6IXD7	Cell division initiation protein DivIVA	125.61	40.09	3.09E+9	39	15	26	4.65	cell cycle; cell division	cytoplasm	Cell division initiation protein DivIVA
F6IYG1	Elongation factor G (EF-G)	75.10	28.51	2.10E+8	15	14	76.9	4.91		cytoplasm	GTPase activity; GTP binding; translation elongation factor activity
G0LZ97	Cell cycle protein GpsB (Guiding PBP1-shuttling protein)	101.39	68.46	7.12E+8	19	11	14.9	7.34	cell cycle; cell division; regulation of cell shape	cytoplasm	Shuttles between the lateral wall and the division site in a cell cycle-dependent manner.
G0M0C4	Uridylate kinase (UK)	52.78	39.58	1.06E+9	16	8	25.9	6.81	de novo' CTP biosynthetic process	cytoplasm	ATP binding; UMP kinase activity
G0M0H0	Elongation factor Tu (EF-Tu)	317.33	67.34	8.25E+9	101	20	43.4	5.05	response to antibiotic;	cytoplasm	GTPase activity; GTP binding; translation elongation factor activity
G0M179	Putative pre-16S rRNA nuclease	41.18	54.17	1.86E+8	8	7	16	5.22	rRNA 5'-end processing	cytoplasm	nuclease activity
G0M3F6	Ribose-phosphate pyrophosphokinase (RPPK)	38.64	23.31	1.62E+8	10	7	35.9	7.25	5-phosphoribose 1-diphosphate biosynthetic process; nucleoside metabolic process; nucleotide biosynthetic process;	cytoplasm	ATP binding; kinase activity; magnesium ion binding;
I8R5D1	Ribosome-binding factor A	28.55	41.03	7.62E+8	9	7	13.1	7.25	maturation of SSU-rRNA	cytoplasm	glucosamine-1-phosphate N-acetyltransferase activity; identical protein
I9ALV0	Elongation factor P (EF-P)	116.55	67.57	6.35E+9	50	12	20.4	5.25		cytoplasm	translation elongation factor activity
I9KZL2	Universal stress protein	119.82	73.91	1.45E+9	18	7	17.7	6.33	response to stress	cytoplasm	adenylate kinase activity
A0A241RP80	Beta sliding clamp	21.03	16.62	2.02E+8	6	6	41.4	4.75	DNA replication	cytoplasm; DNA polymerase III complex	3'-5' exonuclease activity; DNA binding; DNA-directed DNA polymerase activity
I8R888	PTS system, mannose-specific IIB component / PTS system, mannose-specific IIA component	218.97	54.32	5.79E+9	77	17	35.3	6.2	phosphoenolpyruvate-dependent sugar phosphotransferase system	cytoplasm	D-glucosamine PTS permease activity; protein-N(PI)-phosphohistidine-sugar phosphotransferase activity
G0LZW8[r]	Signal recognition particle receptor FtsY (SRP receptor)	40.83	13.25	2.84E+8	8	6	56.9	4.65	SRP-dependent cotranslational protein targeting to membrane	cytoplasm; intrinsic component of plasma membrane	GTPase activity; GTP binding
A0A2K9I1Y7	Cell division protein FtsZ	124.59	33.64	1.77E+9	30	12	45	4.63	cell septum assembly; FtsZ-dependent cytokinesis; protein polymerization	cytoplasm; cell division site	GTPase activity; GTP binding
G0M5H8	2,3-bisphosphoglycerate-dependent phosphoglycerate mutase	121.36	43.48	3.67E+9	35	9	26.1	5.21	gluconeogenesis; glycolytic process	cytoplasm; cytosol	2,3-bisphosphoglycerate-dependent phosphoglycerate mutase activity
I8R8I3	Flavodoxin	18.57	35.81	2.38E+8	4	4	16	4.13	electron transport chain	cytoplasm; cytosol	FMN binding
A0A2I0Z1R6	6-phosphogluconate dehydrogenase, decarboxylating	57.14	37.03	3.22E+8	14	13	53.1	5.2	D-gluconate metabolic process; pentose-phosphate shunt	cytosol	NADP binding; phosphogluconate dehydrogenase (decarboxylating) activity
A0A2I0Z2G5	Pseudouridine synthase	50.01	42.86	3.16E+8	13	9	26.7	9.54	pseudouridine synthesis	cytosol	pseudouridine synthase activity; RNA binding
A0A2I0Z5P0	S-ribosylhomocysteine lyase	13.31	16.46	1.87E+8	5	3	17.4	6.52	quorum sensing	cytosol	iron ion binding; S-ribosylhomocysteine lyase activity
A0A2I0Z612	mRNA interferase	39.72	27.69	5.02E+8	8	3	14.5	10.26	negative regulation of transcription, DNA-templated; negative regulation of translation; quorum sensing; regulation of cell motility	cytosol	DNA binding; endonuclease activity
A0A2K9HY36	Mannitol-1-phosphate 5-dehydrogenase	17.85	10.91	7.51E+7	4	3	43.2	5.27	mannitol metabolic process	cytosol	coenzyme binding; mannitol-1-phosphate 5-dehydrogenase activity
A0A2S9VZ17	Cyclopropane-fatty-acyl-phospholipid synthase	45.86	21.61	1.44E+8	7	6	45.5	6.14	lipid biosynthetic process	cytosol	cyclopropane-fatty-acyl-phospholipid synthase activity
F6IVN7	UTP--glucose-1-phosphate uridylyltransferase	139.41	64.17	1.36E+9	32	15	34.4	6.21	biosynthetic process; UDP-glucose metabolic process	cytosol	UTP:glucose-1-phosphate uridylyltransferase activity
F6IVV5	tRNA/rRNA methyltransferase	20.10	17.24	1.46E+8	4	4	28.1	8.72	RNA processing	cytosol	RNA binding; RNA methyltransferase activity
G0M0C8	D-lactate dehydrogenase (D-LDH)	143.31	63.86	1.93E+9	50	20	37.2	5.06	mixed acid fermentation; response to heat	cytosol	D-lactate dehydrogenase activity; NAD binding
G0M521	2,5-diketo-D-gluconate reductase	80.16	34.55	9.83E+8	18	9	33.4	5.41	L-ascorbic acid biosynthetic process; methylglyoxal catabolic	cytosol	2,5-didehydrogluconate reductase activity

I9KY90	Chromosome partitioning protein, DNA-binding protein	38.37	27.92	4.92E+8	13	1	31.6	8.98	cell division; chromosome condensation; chromosome segregation; DNA replication; sister chromatid cohesion	cytosol	DNA binding [GO:0003677]
A0A2S9WAK8	GMP reductase	86.23	42.82	5.10E+8	17	12	40	5.66	purine nucleotide metabolic process	cytosol; GMP reductase complex	oxidoreductase activity
A0A241RT26	Uracil phosphoribosy-ltransferase	84.48	46.89	4.49E+8	22	9	23	7.49	UMP salvage; uracil salvage	cytosol; membrane	GTP binding; magnesium ion binding; uracil phosphoribosyltransferase activity
G0LZN3	Pyruvate kinase	114.02	39.25	8.73E+8	28	19	62.8	5.14	protein homotetramerization; response to heat	cytosol; membrane	kinase activity; magnesium ion binding; potassium ion binding; pyruvate kinase activity
A0A2S9VPA9	Deoxyuridine 5'-triphosphate nucleotidohydrolase	12.52	17.98	2.34E+8	4	2	19.8	8.84	dUMP biosynthetic process; dUTP catabolic process; dUTP metabolic process	cytosol; protein-containing complex	dUTP diphosphatase activity; magnesium ion binding
A0A2K9I0B1	Catabolite control protein A	29.97	22.32	3.34E+8	9	7	36.3	5.86	regulation of transcription, DNA-templated; transcription, DNA-templated	cytosol; protein-DNA complex	DNA binding
A0A2S9VXT6	50S ribosomal protein L2	245.89	63.44	3.81E+10	265	18	30.1	10.54	translation	large ribosomal subunit	rRNA binding; structural constituent of ribosome; transferase activity
F6IYE2	50S ribosomal protein L15	145.72	64.34	2.20E+10	110	15	15.3	10.64	translation	large ribosomal subunit	rRNA binding; structural constituent of ribosome
A0A241RLS2	30S ribosomal protein S9	135.26	68.46	3.89E+10	65	14	14.6	10.39	translation	ribosome	structural constituent of ribosome
A0A241RM14	50S ribosomal protein L5	113.07	68.89	1.41E+9	29	12	20.2	8.31	translation	ribosome	rRNA binding; structural constituent of ribosome; tRNA binding
A0A241RMV2	50S ribosomal protein L11	104.52	56.74	8.08E+9	38	9	14.8	9.23	translation	ribosome	large ribosomal subunit rRNA binding; structural constituent of ribosome
A0A2I0Z8K7	50S ribosomal protein L3	234.70	55.98	1.67E+10	120	11	22.7	9.99	translation	ribosome	rRNA binding; structural constituent of ribosome
I9L2G2	50S ribosomal protein L4	123.17	54.11	7.02E+9	48	13	22.7	10.15	translation	ribosome	rRNA binding; structural constituent of ribosome
I9L3G1	Ribosome hibernation promoting factor (HPF)	233.73	60.11	7.76E+10	341	12	21.7	5.54	primary metabolic process; regulation of translation	ribosome	ribosomal small subunit binding; ribosome binding
A0A2S9W0G7	30S ribosomal protein S1	436.90	68.76	1.71E+10	179	32	47.2	4.92	translation	ribosome	nucleic acid binding
F6IYC6	50S ribosomal protein L13	205.11	87.50	4.36E+10	90	15	16.8	9.38	translation	ribosome	structural constituent of ribosome
G0M2A8	50S ribosomal protein L23	132.58	77.32	4.38E+10	131	12	11.1	9.66	translation	ribosome	rRNA binding; structural constituent of ribosome
I9L3S1	50S ribosomal protein L7/L12	142.84	93.44	1.27E+10	91	13	12.6	4.48	translation	ribosome	structural constituent of ribosome
A0A2I0Z4F2	Signal recognition protein	72.31	30.58	2.99E+8	14	12	53.8	9.44	SRP-dependent cotranslational protein targeting to membrane	signal recognition particle	7S RNA binding; GTPase activity; GTP binding
A0A241RTU6	30S ribosomal protein S2	334.25	80.52	2.74E+10	185	22	30.2	5.33	translation	small ribosomal subunit	structural constituent of ribosome
G0M2B3	30S ribosomal protein S7	244.76	73.72	1.85E+10	128	18	17.8	9.82	translation	small ribosomal subunit	rRNA binding; structural constituent of ribosome
A0A2I0Z8Q7	30S ribosomal protein S3	104.70	59.91	3.71E+9	42	16	24.2	9.95	translation	small ribosomal subunit	mRNA binding; rRNA binding; structural constituent of ribosome
G0M293	30S ribosomal protein S5	285.01	87.35	2.11E+10	103	18	17.3	9.64	translation	small ribosomal subunit	rRNA binding; structural constituent of ribosome

[a] Protein sequence Uniprot Accession number.
[b] Summary peptides score represents MASCOT score resulting from the ion MS/MS search against the non-redundant Uniprot protein database. All scores are statistically significant ($p<0.05$).
[c] Area represents the relative proportion of each protein.
[d] Theoretical molecular mass.
[e] Isoelectric point.
[f] Protein has a signal peptide.
[g] Protein has Internal transmembrane helices (TMH).

Table 2 Surface-associated proteins of *Lactobacillus. plantarum* HC-2 located on the integral component of the membrane were identified by LC-MS/MS.

Accession [a]	Protein names	Sum PEP Score [b]	Coverage	Area [c]	PSMs	Unique Peptides	MW [kDa] [d]	calc. pI [e]	Gene ontology (biological process)	Gene ontology (cellular component)	Gene ontology (molecular function)
A0A241RM21	Alkaline-shock protein	156.55	64.29	4.94E+9	68	10	15.8	5.03	regulation of transcription, DNA-templated;	integral component of membrane	DNA primase activity; helicase activity; zinc ion binding
A0A241RMK6	TetR/AcrR family transcriptional regulator	18.35	24.61	1.64E+9	9	4	21.6	5.43	regulation of transcription, DNA-[illegible]	integral component of membrane	DNA binding
A0A241RN64	NUDIX domain-containing protein (NUDIX hydrolase)	21.50	36.96	2.06E+8	7	4	15.5	5	biosynthetic process	integral component of membrane	bis(5'-nucleosyl)-tetraphosphatase activity
A0A241RN95	Cof-type HAD-IIB family hydrolase	22.18	23.53	1.36E+8	5	5	31.3	5.05	protein folding	integral component of membrane	hydrolase activity
A0A241RSK0	YtxH domain-containing protein	39.05	21.31	1.40E+9	14	4	19.3	4.3		integral component of membrane	
A0A2I0Z7G6 [f, g]	PDZ domain-containing protein (Serine protease)	114.54	23.81	6.35E+9	30	9	43	9.66	protein catabolic process	integral component of membrane	serine-type endopeptidase activity
A0A2K9HYL7 [f, g]	Glycoside hydrolase family 25	70.05	12.29	3.28E+9	26	8	93.3	9.58	cell wall macromolecule catabolic process; peptidoglycan catabolic process	integral component of membrane	lysozyme activity
A0A2K9I0F3 [g]	PASTA domain-containing protein (Penicillin-binding protein)	35.48	11.50	4.36E+8	8	8	77.2	9.85	cell cycle; cell division; regulation of cell shape	integral component of membrane	penicillin binding
A0A2K9I2I7 [g]	ADP-dependent (S)-NAD(P)H-hydrate dehydratase	16.69	9.29	1.66E+8	5	3	29.8	6.52	nicotinamide nucleotide metabolic process	integral component of membrane	ADP-dependent NAD(P)H-hydrate dehydratase activity; ATP binding
A0A2K9I6Q3	GTP-binding protein TypA	24.93	38.41	6.74E+7	6	5	69.1	5.35	chloroplast RNA processing; regulation of gene expression; response to reactive oxygen species	integral component of membrane	GTPase activity; GTP binding
A0A2K9I7F4 [g]	Peptidase S41	59.12	25.81	4.50E+8	14	11	53.1	9.83	protein catabolic process; proteolysis; response to antibiotic; signal transduction	integral component of membrane	serine-type peptidase activity
A0A2S9VMW3 [g]	Penicillin-binding protein 2	46.55	17.11	3.08E+8	10	7	72.8	9.42	peptidoglycan biosynthetic process	integral component of membrane	penicillin binding
A0A2S9VP39 [g]	LytR family transcriptional regulator	26.55	11.78	1.92E+8	5	4	43.9	9.85	phosphorelay signal transduction system; regulation of transcription, DNA-templated	integral component of membrane	DNA binding

A0A2S9VQT7 [f]	LysM domain-containing protein	23.08	9.76	2.24E+9	12	4	36.9	9.52	cell wall macromolecule catabolic process; peptidoglycan catabolic process	integral component of membrane	lysozyme activity
A0A2S9VQX4	UDP-galactopyranose mutase	23.20	21.51	1.24E+8	6	6	43.2	6.16	Actinobacterium-type cell wall biogenesis; capsule polysaccharide biosynthetic process; cell wall organization	integral component of membrane	UDP-galactopyranose mutase activity
A0A2S9VUM8	Copper oxidase	34.75	20.16	3.06E+8	6	5	56.7	5.03	carbohydrate metabolic process	integral component of membrane	copper ion binding; oxidoreductase activity
A0A2S9VX47	Formate-dependent phosphoribosylglycinamide formyltransferase	40.39	22.70	1.75E+8	7	7	43.1	4.94	'de novo' IMP biosynthetic process	integral component of membrane	ATP binding; metal ion binding
F6IS46 [g]	Integral membrane protein	77.30	51.57	4.01E+8	1	1	14.3	8.63	angiogenesis; cell adhesion; endothelial cell migration;	integral component of membrane	metalloendopeptidase activity; peptidase activity; protease binding; protein dimerization activity; protein homodimerization activity; serine-type
I8R3Q4 [f, g]	Extracellular transglycosylase, with LysM peptidoglycan binding domain	31.54	21.26	2.90E+9	15	4	21.4	8.35	cell wall organization; peptidoglycan biosynthetic process; regulation of cell shape; response to antibiotic	integral component of membrane	penicillin binding; transferase activity
I9KZT8	Mannose-6-phosphate isomerase	14.32	11.84	6.86E+7	4	4	36	5.78	carbohydrate metabolic process	integral component of membrane	mannose-6-phosphate isomerase activity; zinc ion binding
I9L1V1	Phage antirepressor	35.68	25.00	6.59E+8	14	8	29.2	9.1	regulation of transcription, DNA-templated; transcription, DNA-	integral component of membrane	DNA binding
A0A2I0Z060 [g]	ATP-dependent zinc metalloprotease FtsH	63.40	21.21	5.08E+8	13	12	80.8	6.16	protein catabolic process	integral component of membrane	ATPase activity; ATP binding; metalloendopeptidase activity; zinc ion binding
F6IVX9 [g]	PTS family mannose/ fructose/sorbose porter component IID	24.48	11.94	1.66E+8	4	3	34.3	9.39	phosphoenolpyruvate-dependent sugar phosphotransferase system	integral component of membrane	D-glucosamine PTS permease activity; protein-N(PI)-phosphohistidine-sugar phosphotransferase activity
F6IVY0 [g]	Mannose PTS, EIIC	14.95	6.32	8.39E+7	3	1	27.4	5.15	phosphoenolpyruvate-dependent sugar phosphotransferase system	integral component of membrane	protein-N(PI)-phosphohistidine-mannose phosphotransferase system transporter activity
F6IYH9 [g]	Transcription regulator	109.48	45.22	8.18E+8	27	13	37.8	9.98	phosphorelay signal transduction system; positive regulation of DNA-templated tion, initiation	integral component of membrane	identical protein binding; proline -rich region binding; RNA binding; RNA polymerase II repressing transcription factor binding; transcription
G0M2X1 [g]	Phosphate ABC superfamily ATP binding cassette transporter, binding protein	15.23	9.06	7.60E+7	2	2	32.5	9.94	phosphate ion transmembrane transport; phosphate ion transport; regulation of phosphatase activity	integral component of membrane	phosphate ion binding
I8RA29 [g]	Transpeptidase-transglycosylase (Penicillin binding protein 1A)	84.07	21.92	4.72E+8	18	13	83.5	9.38	cell wall organization; peptidoglycan biosynthetic process; regulation of cell shape	integral component of membrane	penicillin binding; transferase activity
A0A241RN35 [f, g]	Glutamine ABC transporter substrate-binding protein	63.65	42.09	4.67E+8	14	10	30	9.7	nitrogen compound transport	integral component of membrane	ionotropic glutamate receptor activity
A0A2K9IAS1	Glycine/betaine ABC transporter ATP-binding protein	34.76	26.88	9.43E+7	9	9	44.4	5.21	glycine betaine transport	integral component of membrane	ATPase activity; ATP binding
A0A2S9WBP2 [f]	Maltodextrin-binding protein	138.12	39.00	1.92E+9	33	15	45.7	9.66	carbohydrate transport; cell chemotaxis; cellular response to DNA damage stimulus; detection of maltose stimulus;	plasma membrane	carbohydrate transmembrane transporter activity
I9AMS3 [f]	Foldase protein PrsA	129.60	42.62	7.29E+9	48	18	32.7	9.73	protein folding	plasma membrane	peptidyl-prolyl cis-trans isomerase activity
A0A241RS35	ATP synthase subunit alpha	62.93	21.15	8.81E+8	16	10	54.7	5.1	ATP synthesis coupled proton transport	plasma membrane; proton-transporting ATP synthase complex, catalytic core F(1)	ATP binding; proton-transporting ATP synthase activity, rotational mechanism
A0A2K9I151	ATP synthase subunit beta (F-ATPase subunit beta)	60.71	28.05	3.26E+8	10	9	50.8	4.72	ATP synthesis coupled proton transport	plasma membrane; proton-transporting ATP synthase complex, catalytic core F(1)	ATP binding; proton-transporting ATP synthase activity, rotational mechanism
F6ISI9 [f, g]	ABC superfamily ATP binding cassette transporter, binding protein	30.06	22.12	1.59E+8	7	6	35.8	9.74	response to antibiotic	integral component of membrane; plasma membrane	ATP binding; lipid-transporting ATPase activity
A0A2I0Z1P7 [g]	Endolytic murein transglycosylase (Peptidoglycan polymerization terminase)	36.99	17.46	2.97E+8	8	6	44.7	9.7	cell wall organization; peptidoglycan biosynthetic process	integral component of plasma membrane	lyase activity; lytic endotransglycosylase activity
G0M1N0 [f, g]	Cell shape-determining protein MreC (Cell shape protein MreC)	20.13	15.66	3.71E+8	6	4	30.1	9.67	regulation of cell shape	integral component of plasma membrane	protein self-association
A0A2K9I4I2 [f]	FAD:protein FMN transferase (Flavin transferase)	44.33	26.49	1.90E+8	8	7	40.4	9.69	protein flavinylation	integral component of membrane; plasma membrane	metal ion binding; transferase activity
A0A2K9I4P5	Peptidase M13	29.32	10.50	1.86E+8	6	5	71.8	5.15	proteolysis in other organism	integral component of membrane; plasma membrane	metalloendopeptidase activity
A0A2S9VLD0 [f]	Hydrolase	30.85	16.67	3.93E+8	6	4	31.9	9.64	cell wall organization	integral component of membrane; plasma membrane	hydrolase activity
F6IYY1	Negative regulator of proteolysis	62.40	50.00	1.51E+9	20	8	16.3	6.2	bacterial-type flagellum organization; negative regulation of proteolysis; negative regulation of transcription, DNA-templated;	integral component of membrane; plasma membrane	sigma factor antagonist activity

[a] Protein sequence Uniprot Accession number.
[b] Summary peptides score represents MASCOT score resulting from the ion MS/MS search against the non-redundant Uniprot protein database. All scores are statistically significant ($p<0.05$).
[c] Area represents the relative proportion of each protein.
[d] Theoretical molecular mass.
[e] Isoelectric point.
[f] Protein has a signal peptide.
[g] Protein has Internal transmembrane helices (TMH).

Table 3. Surface-associated proteins of *Lactobacillus. plantarum* HC-2 located on cell surface/cell wall were identified by LC-MS/MS.

Accession [a]	Protein names	Sum PEP Score [b]	Coverage	Area [c]	PSMs	Unique Peptides	MW [kDa] [d]	calc. pI [e]	Gene ontology (biological process)	Gene ontology (cellular component)	Gene ontology (molecular function)
G0M222 [f]	Lipoprotein	104.71	50.64	3.03E+8	15	12	42.5	9.38	growth of symbiont in host; modulation by symbiont of host immune response; modulation by symbiont of microbe-associated molecular pattern-induced host innate immune response;	bacterial extracellular vesicle; cell surface; cell wall; host cell cytoplasm; plasma membrane	host cell surface receptor binding

									pathogenesis; positive regulation by symbiont of host apoptotic process		
A0A2I0Z8V7 [f, g]	Metal ABC transporter substrate-binding protein	7.45	7.35	5.15E+7	2	2	34.8	9.51	cell adhesion ; metal ion transport	cell outer membrane; cell surface	metal ion binding
A0A2I0Z8X9	GatB/YqeY domain-containing protein	30.80	41.50	7.62E+8	9	6	16.1	6.2		cell outer membrane; integral component of membrane	carbon-nitrogen ligase activity, with glutamine as amido-N-donor
A0A2K9IAR0	Protein phosphatase	39.07	13.31	3.70E+8	6	2	27.2	4.79	glycerophospholipid biosynthetic process; peptidoglycan biosynthetic process; phosphatidylglycerol biosynthetic process; phospholipid catabolic process	cell outer membrane; integral component of plasma membrane; plasma membrane	protein serine/threonine phosphatase activity
G0M0A2	Chaperone protein DnaK (HSP70)	504.65	64.31	1.76E+10	159	32	66.7	4.82	protein folding	cell surface	ATP binding; unfolded protein binding
G0M2S6	Enolase (2-phospho-D-glycerate hydro-lyase) (2-phosphoglycerate dehydratase)	161.31	53.85	2.28E+9	53	19	48	4.74	glycolytic process	cell surface; extracellular region; phosphopyruvate hydratase complex	magnesium ion binding; phosphopyruvate hydratase activity
A0A2K9HZK7 [f]	Cell wall hydrolase	83.55	9.86	3.52E+10	48	7	82.8	9.16	cell wall organization	cell surface; extracellular region; fungal-type cell wall; hyphal cell wall	amidase activity
A0A2I0Z4N9 [f, g]	Cell surface protein	41.07	7.50	3.43E+9	17	5	65.3	4.37	cell adhesion; metal ion transport	cell wall	collagen binding
A0A2I0Z1G1	Dihydrolipoamide acetyltransferase component of pyruvate dehydrogenase complex	54.13	23.69	3.77E+8	11	7	46.7	4.96	metabolic process	cell wall; cytosol; plasma membrane; pyruvate dehydrogenase complex	transferase activity, transferring acyl groups
G0LYK7 [f]	Extracellular protein	43.60	17.65	2.40E+9	9	3	24.6	9.38	carbohydrate metabolic process; carbohydrate utilization	cell wall; extracellular region	carbohydrate metabolic process; carbohydrate utilization
I9KX18 [f]	Extracellular protein, gamma-D-glutamate-meso-diaminopimelate muropeptidase	58.36	56.63	1.65E+10	14	1	37.3	9.38	cell wall organization	cell wall; extracellular region	cysteine-type peptidase activity
I9KYQ3 [f, g]	Extracellular transglycosylase, membrane-bound	61.97	70.61	1.58E+8	2	1	15.4	10.48	cell wall organization; peptidoglycan turnover	cell wall; extracellular region	hydrolase activity, hydrolyzing O-glycosyl compounds; lyase activity
A0A2S9W1F0 [f]	Mucus-binding protein	121.95	60.77	7.98E+7	1	1	141.1	5.21	cell adhesion	cell wall; extracellular region; integral component of membrane	
A0A2I0Z5P8 [f]	LysM peptidoglycan-binding domain-containing protein	21.82	8.60	2.54E+10	25	2	36.8	9.26	pathogenesis;peptidoglycan metabolic process	cell wall; extracellular region; membrane	
G0M2R3	ATP/GTP hydrolase	36.72	30.19	7.62E+8	11	4	17.7	4.97	tRNA threonylcarbamoyladenosine modification	cell wall; plasma membrane	hydrolase activity

[a] Protein sequence Uniprot Accession number.

[b] Summary peptides score represents MASCOT score resulting from the ion MS/MS search against the non-redundant Uniprot protein database. All scores are statistically significant (p<0.05).

[c] Area represents the relative proportion of each protein.

[d] Theoretical molecular mass.

[e] Isoelectric point.

[f] Protein has a signal peptide.

[g] Protein has Internal transmembrane helices (TMH).

Table 4. Surface-associated proteins of *Lactobacillus. plantarum* HC-2 located on cells or others were identified by LC-MS/MS.

Accession [a]	Protein names	Sum PEP Score [b]	Coverage	Area [c]	PSMs	Unique Peptides	MW [kDa] [d]	calc. pI [e]	Gene ontology (biological process)	Gene ontology (cellular component)	Gene ontology (molecular function)
A0A241RQI8 [f]	Amino acid ABC transporter substrate-binding protein	71.25	25.56	6.44E+8	12	2	28.8	10.02	nitrogen compound transport	ATP-binding cassette (ABC) transporter complex	metal ion binding
A0A2S9W6Z6 [f]	Peptide ABC transporter substrate-binding protein	67.73	24.55	7.52E+8	22	4	61	9.72	transmembrane transport	ATP-binding cassette (ABC) transporter complex	
G0M1I4 [f]	Oligopeptide ABC transporter, substrate binding protein	164.06	39.96	1.06E+9	45	8	60.3	9.63	transmembrane transport	ATP-binding cassette (ABC) transporter complex	
A0A241RS75	Thiol peroxidase	38.86	26.22	1.97E+9	7	2	18.2	5.54	cell redox homeostasis	cell	thioredoxin peroxidase activity
A0A241RTI6	DNA starvation/stationary phase protection protein	105.26	71.61	1.69E+10	50	10	18	4.79	cellular iron ion homeostasis; response to stress	cell	ferric iron binding; oxidoreductase activity, oxidizing metal ions
I8R8J3	Thioredoxin H-type	27.61	58.49	8.98E+8	6	4	12.3	4.7	cell redox homeostasis; glycerol ether metabolic process	cell	protein disulfide oxidoreductase activity
I8R7W6	Glutathione reductase	139.55	23.20	1.03E+10	58	2	48.5	4.94	cell redox homeostasis	cell	electron transfer activity; flavin adenine dinucleotide binding
F6IYZ2	Putative transcription regulator	11.26	14.71	4.64E+7	3	2	19.7	9.19	transcription, DNA-templated	intracellular	DNA binding; DNA-binding transcription factor activity
G0M4C7	Response regulator	144.55	40.43	2.01E+9	33	10	27	5.34	phosphorelay signal transduction system; regulation of transcription, DNA-templated	intracellular	DNA binding
I8RAE9	Two-component system response regulator	29.10	25.44	3.63E+8	10	7	26.4	5.16	phosphorelay signal transduction system; regulation of transcription, DNA-templated	intracellular	DNA binding
A0A241RS46	Glycine cleavage system protein H	18.38	30.61	4.70E+8	4	3	10.5	4.53	glycine decarboxylation via glycine cleavage system	mitochondrion	dihydrolipoyl dehydrogenase activity; disulfide oxidoredu -ctase activity; electron transfer activity; flavin adenine dinucleotide binding; binding protein
A0A120MH48	Heat shock protein 70 (Fragment)	156.78	74.53	8.43E+8	48	1	22.5	5	protein folding		ATP binding
A0A241RKI5	Enoyl-[acyl-carrier-protein] reductase [NADH]	50.38	29.37	2.74E+8	11	6	26.9	6.21	fatty acid biosynthetic process		enoyl-[acyl-carrier-protein] reductase (NADH) activity; enoyl-[acyl-carrier-protein] reductase activity
A0A2K9I0D1	Adapter protein MecA	42.93	30.86	2.57E+8	9	7	27.8	4.55	establishment of competence for transformation; negative regulation of establishment of competence for transformation; negative regulation of sporulation resulting in formation of a cellular spore; sporulation resulting in formation of a cellular spore		protein binding, bridging
A0A2K9I4H9	Phosphate acetyltransferase	31.10	23.08	4.24E+8	6	5	34.4	5.45	metabolic process		acetyltransferase activity

A0A2S9VMB1	Phosphoketolase	65.43	19.04	2.54E+8	14	11	88.7	5.27	carbohydrate metabolic process	aldehyde-lyase activity
A0A2S9VSN5	Single-stranded DNA-binding protein	30.25	15.63	6.35E+8	10	6	38.9	8.65	DNA recombination; DNA repair; DNA replication	DNA binding
A0A2S9VX41	Aspartyl/glutamyl-tRNA (Asn/Gln) amidotransferase subunit C	17.21	29.25	6.65E+7	2	2	11.6	4.59	regulation of translational fidelity; translation	glutaminyl-tRNA synthase (glutamine-hydrolyzing) activity; transferase activity
A0A2S9W7K7	Phosphoglucomutase	25.84	13.57	1.64E+8	7	7	63.6	5.06	carbohydrate metabolic process	magnesium ion binding; phosphoglucomutase activity
F6ISC3	Transcription antitermination protein NusB	35.96	28.06	1.52E+9	10	5	15.7	4.93	DNA-templated transcription, termination; transcription antitermination	RNA binding
F6ISE9	Transcription elongation factor GreA	149.29	81.88	2.81E+9	27	10	17.9	4.67	regulation of DNA-templated transcription, elongation; transcription, DNA-templated	DNA binding; RNA polymerase binding; translation elongation factor activity
F6ITJ9	Fructose-bisphosphate aldolase	141.17	47.74	3.36E+9	50	13	30.9	5.12	fructose 1,6-bisphosphate metabolic process; glycolytic process	fructose-bisphosphate aldolase activity; zinc ion binding
F6IUH8	Single-stranded DNA-binding protein (SSB)	39.20	26.90	5.53E+8	10	6	21.4	5.12	DNA recombination; DNA repair; DNA replication	single-stranded DNA binding
F6IVG6	Transcriptional repressor NrdR	14.02	16.37	4.54E+8	2	2	19.9	8.44	negative regulation of transcription, DNA-templated; transcription, DNA-templated	ATP binding; DNA binding; zinc ion binding
F6IVV3	Transcription termination/antitermination protein NusG	34.16	29.12	5.84E+8	10	5	20.6	4.83	DNA-templated transcription, elongation;	
F6IXY9	Transcriptional regulator	16.41	15.84	2.55E+8	4	4	24.6	5.3	regulation of transcription, DNA-templated; transcription, DNA-templated	DNA binding
F6IZ27	ATP-dependent Clp protease, ATP-binding subunit ClpL	160.74	35.85	7.33E+8	38	22	75.9	5.35	regulation of transcription, DNA-templated; transcription, DNA-templated	ATP binding; peptidase activity
G0LYW7	Bifunctional protein PyrR	209.02	83.89	9.86E+9	103	13	19.8	6	DNA-templated transcription, termination; nucleoside metabolic process; regulation of transcription, DNA-templated	RNA binding; uracil phosphoribosyltransferase activity
G0LZX8	Ribulose-phosphate 3-epimerase	34.57	20.28	6.95E+8	10	4	23.4	5.1	carbohydrate metabolic process; pentose-phosphate shunt	metal ion binding; ribulose-phosphate 3-epimerase activity
G0M0E9	Tagatose-6-phosphate kinase	20.82	14.43	1.79E+8	3	3	32.4	5.15	D-tagatose 6-phosphate catabolic process; lactose metabolic process	1-phosphofructokinase activity; ATP binding
G0M1A8	Phage-related major head protein	7.77	7.37	6.64E+7	3	1	41.1	5.1		
G0M392	N-acetylglucosamine-6-phosphate deacetylase	46.88	24.87	4.98E+8	12	10	41.5	5.27	carbohydrate metabolic process; N-acetylglucosamine metabolic process	metal ion binding; N-acetylglucosamine-6-phosphate deacetylase activity
G0M3X1	Nucleoside-diphosphate kinase	74.79	57.79	2.17E+9	25	7	17	8.81	CTP biosynthetic process; GTP biosynthetic process; UTP biosynthetic process	nucleoside diphosphate kinase activity
G0M6A2 [f]	Cellobiose PTS, EIIB (EC 2.7.1.69)	116.66	71.43	4.08E+9	37	7	11.3	4.75	phosphoenolpyruvate-dependent sugar phosphotransferase system	D-glucosamine PTS permease activity; protein-N(PI)-phosphohistidine-sugar phosphotransferase activity
I8R539	Peptidyl-prolyl cis-trans isomerase (PPIase) (EC 5.2.1.8)	63.66	27.32	3.51E+8	15	5	21.1	4.7		peptidyl-prolyl cis-trans isomerase activity

[a] Protein sequence Uniprot Accession number.
[b] Summary peptides score represents MASCOT score resulting from the ion MS/MS search against the non-redundant Uniprot protein database. All scores are statistically significant ($p<0.05$).
[c] Area represents the relative proportion of each protein.
[d] Theoretical molecular mass.
[e] Isoelectric point.
[f] Protein has a signal peptide.
[g] Protein has Internal transmembrane helices (TMH).
